# Parental energy-sensing pathways control intergenerational offspring sex determination in the nematode *Auanema freiburgensis*

**DOI:** 10.1186/s12915-021-01032-1

**Published:** 2021-05-17

**Authors:** Pedro Robles, Anisa Turner, Giusy Zuco, Sally Adams, Panagiota Paganopolou, Michael Winton, Beth Hill, Vikas Kache, Christine Bateson, Andre Pires-daSilva

**Affiliations:** 1 School of Life Sciences, University of Warwick, Coventry, CV4 7AL UK; 2 Department of Biology, University of Texas at Arlington, Arlington, TX 76019 USA

**Keywords:** Intergenerational inheritance, Transgenerational inheritance, Dauer, Sex determination, nematode, *C. elegans*, *Auanema*, AMPK, Histone acetylation

## Abstract

**Background:**

Environmental stimuli experienced by the parental generation influence the phenotype of subsequent generations (Demoinet et al., Proc Natl Acad Sci U S A 114:E2689-E2698, 2017; Burton et al., Nat Cell Biol 19:252–257, 2017; Agrawal et al., Nature 401:60-63, 1999). The effects of these stimuli on the parental generation may be passed through the germline, but the mechanisms at the basis of this non-Mendelian type of inheritance, their level of conservation, how they lead to adaptive vs non-adaptive, and intergenerational vs transgenerational inheritance are poorly understood. Here we show that modulation of nutrient-sensing pathways in the parental generation of the nematode *Auanema freiburgensis* regulates phenotypic plasticity of its offspring.

**Results:**

In response to con-specific pheromones indicative of stress, AMP-activated protein kinase (AMPK), mechanistic target of rapamycin complex 1 (mTORC1), and insulin signaling regulate stress resistance and sex determination across one generation, and these effects can be mimicked by pathway modulators. The effectors of these pathways are closely associated with the chromatin, and their regulation affects the chromatin acetylation status in the germline.

**Conclusion:**

These results suggest that highly conserved metabolic sensors regulate phenotypic plasticity through regulation of subcellular localization of their effectors, leading to changes in chromatin acetylation and epigenetic status of the germline.

**Supplementary Information:**

The online version contains supplementary material available at 10.1186/s12915-021-01032-1.

## Background

The phenotype of an individual is the result of the interactions between its genome and the environment. Experiences made by the ancestors may also shape an individual’s phenotype. Some of those traits are transmitted through the germline, including mechanisms that do not involve changes in DNA sequence [1, 2]. Some recent examples of traits that are “epigenetically” inherited include immunity [3, 4], behavior [5, 6], resistance to starvation [7], and osmotic stress [8].

To understand the mechanisms of epigenetically inherited traits, it is important to distinguish between transgenerational and intergenerational inheritance [9]. Transgenerational effects refer to information that is passed across multiple generations and that affect the phenotype even when the stimulus is absent. Intergenerational effects refer to parental exposures affecting primarily the phenotype of the immediate generation. These effects can be passed either through the germline or the soma [10]. Recently, the mechanisms underlying gametic transmission of parental effects have been revealed in some animal models. The germ cells of parents fed on specific diets, for example, may undergo chromatin modifications [11], changes in the composition of fragments of tRNAs [12, 13] and yolk content [14, 15] that are consequential for the phenotype of the offspring. While these mechanisms seem passive effects of the parental experiences and may not always be adaptive, there are known examples for intergenerational adaptive responses to the environment [16, 17]. In the crustacean *Daphnia*, for example, chemical sensing of predators by the parents induces the production of predation-resistant offspring [18].

The nematode *Caenorhabditis elegans* has been instrumental in revealing mechanisms of inter- and transgenerational inheritance because of its short generation time, large number of offspring, and availability of genetic resources. While transgenerational effects are superficially mediated by similar mechanisms as for intergenerational effects in this nematode, such as chromatin modifications [19] and small RNAs [20], many questions still remain: what are the mechanisms that determine whether traits are transmitted for either one or multiple generations? How general are these mechanisms across nematodes and the animal kingdom? Are there differences in mechanisms when traits are transmitted from somatic cells to the germline, versus environmental cues that act directly on the germline? Are there differences in mechanisms that result in adaptive versus non-adaptive traits?

To address some of these questions, we have been studying *Auanema* nematodes. Similar to *C. elegans*, these nematodes have a short generation time (~ 4 days) and produce a large number (~ 350) of offspring [21, 22]. Nematodes of this genus have three sexual morphs [23]: males are XO, whereas hermaphrodites and females are XX [24]. In *A. rhodensis*, the maternal environment seems important for the hermaphrodite versus female fate, since most females are produced by young mothers, whereas older mothers produce mostly self-fertilizing hermaphrodites [22]. Although phenotypically almost identical at the adult stage, the larval development is different between *Auanema* hermaphrodites and females: hermaphrodites always develop through a starvation-resistant larval stage named “dauer”. In fact, dauer development is determinant for the sexual morph fate, since larvae initially committed to become females can be converted to hermaphrodites if forced to undergo dauer formation [25].

Here we focus on the species *Auanema freiburgensis*, for which the environmental cues experienced by the mother determine dauer formation and sexual morph fate of the offspring [23]. We show that signals indicative of crowding can induce the mother to produce dauers, which become hermaphrodite adults. Both traits (stress resistance and sexual morph fate) are likely to be adaptive and inherited intergenerationally by the transmission of signals sensed by somatic cells to germline. Pharmacological assays indicate that energy-sensing signaling mediated by AMP-activated protein kinase (AMPK), mechanistic target of rapamycin complex 1 (mTORC1), and insulin signaling are involved in intergenerational inheritance in *A. freiburgensis*. The formation of F1 dauers correlates with changes in the germline histone acetylation of the maternal germline.

## Results

**Fig. 1.**
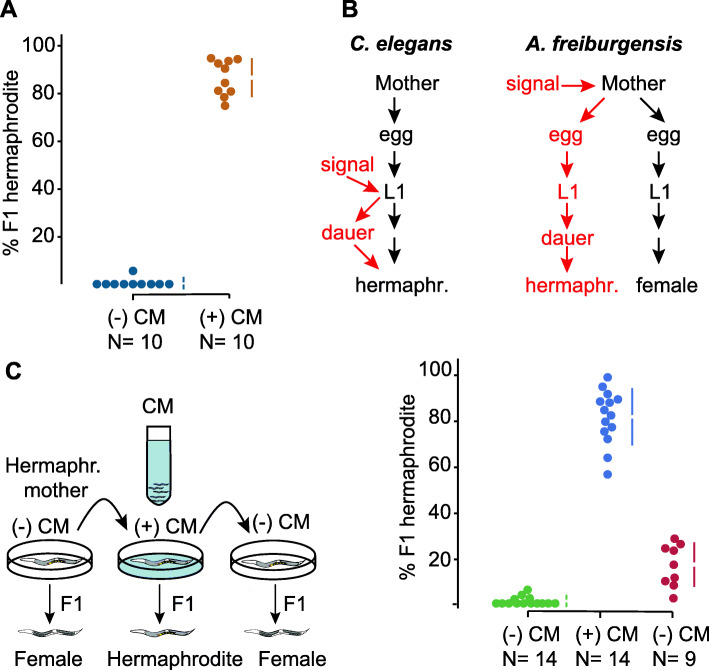
Dauer and hermaphrodite development are induced across generations in *A. freiburgensis*. **a** When hermaphrodite mothers are cultured in non-crowding conditions, in the absence of conditioned medium (CM), most of the XX F1s are female (*N* = 10 broods, from which a total of 149 F1s were scored). When mothers are in CM of crowded cultures, most of the XX F1s are hermaphrodites (*N* = 10 broods, with a total of 199 F1s scored). The data in colored dots represent the percentage of F1 hermaphrodites in each brood and is plotted on the upper axes. The colored vertical lines indicate ± SD, and the mean is represented as a gap in the lines. **b** In *C. elegans*, the L1 larvae respond to environmental signals to facultatively form stress-resistant dauers. In *A. freiburgensis*, it is the mother and not the L1s that respond to environmental signals. *A. freiburgensis* dauers obligatorily develop into hermaphrodite adults. **c** In the experimental setup (top), the same individual mother hermaphrodite was transferred every 24 h to a new environmental condition. Initially, it was placed in a plate without conditioned medium (−) CM, followed by the transfer to a (+) CM plate and then to a new (−) CM plate. The plot representation is the same as for Fig. 1a. On the last day, 5 mothers died and thus only 9 broods were scored

Individuals of the nematode *A. freiburgensis* produce only sperm (males), only oocytes (females), or both gametes (hermaphrodites) [23]. The hermaphrodite versus female sexual morph is determined by the environment experienced by the mother: hermaphrodite mothers kept in isolation produce mostly female offspring, whereas hermaphrodites exposed to high population density conditions produce mostly hermaphrodite offspring (Fig. 1a).

A crucial factor in the development of *Auanema* hermaphrodites is the passage through the stress-resistant dauer stage [21–23, 25], which has morphological and behavioral adaptations for dispersal. In *A. freiburgensis*, all XX larvae that pass through the dauer stage become hermaphrodites (*N* = 96), whereas XX non-dauer larvae develop into females (*N* = 93). Similar to *A. rhodensis* [25] and other trioecious nematodes [26], we never observed *A. freiburgensis* males to undergo dauer formation. Thus, signals experienced by the maternal generation of *A. freiburgensis* are used to generate non-feeding offspring that can survive starvation conditions and reproduce by self-fertilization once food becomes available. In summary, these results suggest that dauer formation in *A. freiburgensis* is induced across a generation, instead of within the same generation as in *C. elegans* [27] (Fig. 1b).

High population density conditions were induced by incubating *A. freiburgensis* hermaphrodites with conditioned medium (CM) derived from liquid cultures containing high nematode population densities (see “Methods”). Importantly, only the parental generation was exposed to the CM. The induction of dauers through the hermaphrodite mother is limited to one generation: F1 hermaphrodites derived from mothers in (+) CM plates produce mostly female offspring (99.6% out of 470 F2 offspring, scored from 10 broods). To test if *A. freiburgensis* L1 larvae can also respond to crowding conditions, eggs derived from mothers cultured in isolation were left to hatch and undergo larval development in (+) CM plates until adulthood. 95.7% (*N* = 161) of these L1s developed into females, indicating the effects of crowding are detected only in the parental generation.

To test if the effects of the crowding conditions on the hermaphrodites acted in a reversible manner, the same individual was placed in different conditions on different days. When an individual was in non-crowded conditions, most of the offspring was composed of females (Fig. 1c). When the same individual was placed in plates with the crowding signal, it produced mostly hermaphrodites. After rinsing it with the M9 buffer and placing the same individual in a plate with no crowding cues, it resumed the production of females. These results suggest that a hermaphrodite adjusts the production of sexual morphs according to the immediate environmental signals.

To determine the minimal population density sufficient for the induction of dauers and hermaphrodites across a generation, we incubated the maternal generation at different densities. When hermaphrodites are cultured for 6 h, a minimum density of 16 adult hermaphrodites per cm^2^ is sufficient for the induction of 100% (*N* = 295 F1s) of hermaphrodite offspring. In densities below 10 individuals/cm^2^, the hermaphrodite mothers produce only female offspring (10 individuals/cm^2^: 100% females, *N* = 78 F1s; 6 individuals/cm^2^: 98.5% F1 female, *N* = 66 F1s). At an intermediate density (13 individuals/cm^2^), hermaphrodites produce 19% (*N* = 126 F1s) of hermaphrodite offspring.

To investigate if other maternal environmental conditions affect the sexual fate of the F1s, mothers were incubated for 24 h at 25 °C or subjected to diet restriction conditions (no food). Most XX offspring (97%) developed into female adults for both conditions (166 F1s scored from mothers at 25 °C and 146 F1s scored from starving mothers).

### Modulation of AMPK signaling changes sexual morph ratios in *A. freiburgensis*

In previous research, energy metabolism has been associated with intergenerational and transgenerational inheritance in *C. elegans* [20, 28, 29]. Although *A. freiburgensis* subjected to diet restriction did not induce dauer formation in F1s, it is possible that the high-crowding conditions experienced by the parental generation are used as indication for imminent lack of food. In eukaryotes, caloric restriction triggers the activation of AMP-activated protein kinase (AMPK) [30], a highly conserved energy sensor [31]. AMPK activity protects cells against the depletion of ATP by stimulating energy-producing pathways and inhibiting energy-consuming processes [32]. In *C. elegans*, AMPK is required for lifespan extension and germline viability when the nematode is in nutrient stress [28, 30, 33, 34]. The full kinase activity of AMPK requires phosphorylation of threonine residue 172 (Thr172) by upstream kinases [30, 35, 36].

**Fig. 2.**
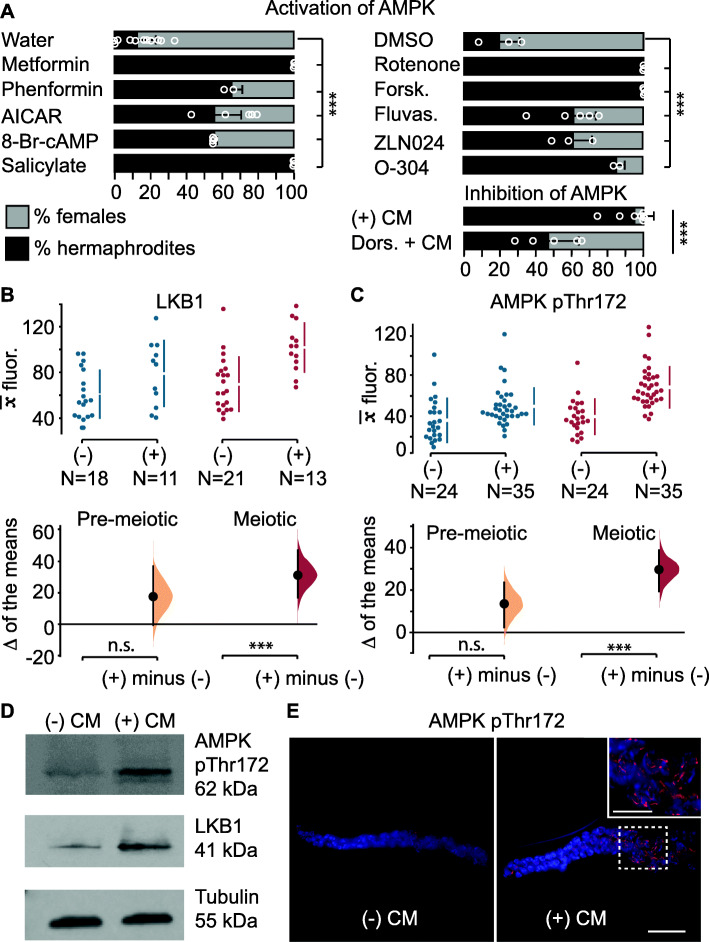
AMPK pathway modulation in the *A. freiburgensis* germline. **a** Mean percentage of hermaphrodite and female F1 offspring from hermaphrodites treated with chemicals. The control was either water (left) or DMSO (right), depending on how the chemical compounds were dissolved. Error bars represent SD. Dors. = Dorsomorphin. In all cases, diluted (1:10) CM was added to the medium, with exception to plates with dorsomorphin, which had undiluted CM. **b, c** Mean antibody fluorescence () in the premeiotic (blue) and meiotic portion (red) of the germline, in the absence (−) or presence (+) of conditioned medium. *N* = sample sizes. The mean difference for the two comparisons is shown as a Gardman-Altman estimation plot. The raw data is plotted on the upper axes, with colored vertical lines indicating ± 95% CI, and the mean is represented as a gap in the lines. Each difference of the means is plotted on the lower axes as a bootstrap sampling distribution. The difference of the means is depicted as a black dot and 95% confidence intervals are indicated by the black vertical error bars. n.s., *p* > 0.05; *** = *p* ≤ 0.01. **d** Western blots with proteins derived from hermaphrodites incubated in the absence (−) CM or presence (+) CM of conditioned medium. **e** AMPK pThr172 antibody staining of gonads dissected from hermaphrodites incubated in either (−) CM or (+) CM. Bar, 15 μm. Insert in the right picture is a magnification from the region marked with a stippled square. Bar, 7.5 μm. Details for the one-way analysis of variance (ANOVA) followed by a post hoc Holm-Sidak test for multiple comparisons (**a**), *** = *p* ≤ 0.01. See “Methods” for sample sizes and *p* values.

We hypothesized that AMPK regulates target proteins in the maternal germline to influence the phenotype of the following generation. To functionally test the role of AMPK in mediating intergenerational inheritance in *A. freiburgensis*, we used pharmacological compounds that modulate AMPK activity. We measured the effects of these compounds on intergenerational inheritance by scoring hermaphrodite and female sexual morphs in the offspring. As mentioned previously, high population densities induce the production of dauer larvae in the F1, which mature to become hermaphrodite adults. Consistent with a role of AMPK in mediating this effect, we found that AMPK activators induce the production of hermaphrodites (Fig. 2a) (for a recent review on pharmacological activation of AMPK, see [37]). In some cases, these compounds cause changes in the F1 sex morph ratios when on their own (Additional file 1: Figure S2), but potentiation of their effects was significantly stronger when diluted CM (1:10 CM) was added to the culture medium. This may indicate that synergistic effects of different mechanisms are necessary to fully elicit a robust response, or that those energy-sensing pathways can be efficiently activated only when upstream events occur first.

Although the mechanisms of action are not clear for all pharmacological compounds, they can be broadly divided into indirect and direct AMPK activators. Any treatments that raise the AMP/ADP:ATP ratios are expected to indirectly activate AMPK. For instance, inhibition of mitochondrial respiration by metformin, phenformin, and rotenone has been implicated in the activation of AMPK [38–45]. Forskolin, an adenylate cyclase activator, activates AMPK by increasing the cytosolic cAMP concentration [46]. Statins, such as fluvastatin [47], have been proposed to activate AMPK. The incubation of mothers with all these compounds resulted in a higher proportion of hermaphrodite progeny (Fig. 2a).

Compounds that are similar to AMP can directly activate AMPK. 5-Aminoimidazole-4-carboxamide ribonucleotide (AICAR), for example, increases the activity of AMPK after being converted to an AMP analog inside the cell [48], whereas 8-Br-cAMP is a non-hydrolyzable analog of cAMP [49]. Other compounds, such as the plant product salicylate [50], and the synthetic compounds ZLN024 [51] and O-304 [52] bind to AMPK, causing allosteric activation and inhibition of dephosphorylation of the pThr172. All these compounds induced a higher percentage of hermaphrodite offspring than controls (Fig. 2a). When tested at various concentrations, metformin induced hermaphrodites in a dose-dependent manner (Additional file 1: Figure S2B). To inhibit AMPK, we used dorsomorphin [40]. As expected, hermaphrodites in CM with dorsomorphin resulted in a lower proportion of hermaphrodite progeny compared to controls (Fig. 2a).

To examine if levels of AMPK activity in the change according to crowding conditions, we performed antibody staining on dissected gonads and Western blots of whole animals (Fig. 2b–e). Liver Kinase B1 (LKB1), known in *C. elegans* as PAR-4 [36, 53], phosphorylates and activates AMPK in the context of energy stress [54, 55]. LKB1 is part of a complex with two proteins Ste20-related adaptor protein-alpha (STRAD) [56] and mouse protein 25-alpha (MO25alpha) [57]. Antibody staining against LKB1 and STRAD showed a higher level of staining in the meiotic portion of the germline isolated from animals cultured in crowding conditions (Fig. 2b, d and Additional file 1: Figure S1). Their localization was predominant in the cytoplasm of germline cells (Additional file 1: Figure S1).

To test the levels of AMPK, we used an antibody that detects the active, phosphorylated form of AMPK (AMPK pThr172). Consistent with the higher levels of LKB1 and STRAD, we also found that the anti-AMPK pThr172 staining was stronger in crowding conditions compared to control animals (Fig. 2c–e). The difference in the level of staining was restricted to the meiotic region of the germline and the AMPK staining is closely associated with the chromatin of pachytene cells (Fig. 2e).

### Maternal inhibition of mTORC1 signaling results in mostly hermaphrodite offspring

Since energy-sensing by AMPK induced intergenerational effects in *A. freiburgensis*, we hypothesized that other energy sensors may be involved in the same process. The intracellular nutrient sensor mTORC1 complex is a multisubunit kinase that senses growth signals and stimulates anabolism when nutrients are abundant [58–62]. Therefore, we would predict that in low population densities and readily available nutrients, the mTOR pathway would be active in *A. freiburgensis*. Under these conditions, *A. freiburgensis* produces mostly non-dauer larvae that later become female offspring.

**Fig. 3.**
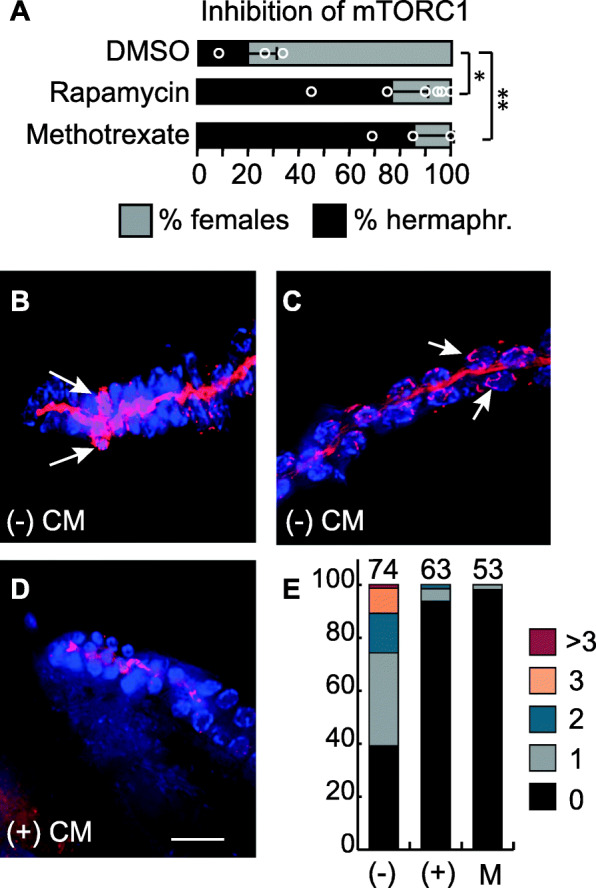
mTOR signaling modulates intergenerational inheritance in *A. freiburgensis*. **a** Mean percentage and SD of hermaphrodite and female F1 offspring from hermaphrodites incubated with either DMSO or pharmacological compounds, together with some CM (1:10) CM. One-way analysis of variance (ANOVA). *p ≤ 0.05, **p ≤ 0.01. **b–d** Staining with S6K pThr389 antibody (red) and DAPI (blue) of dissected gonads from hermaphrodites incubated either in the absence ((−) CM) or in the presence ((+) CM) of conditioned medium. The arrows indicate cells marked with the antibody in the premeiotic region (**b**) and in the meiotic region (**c**), respectively. In **d**, only the rachis has staining. Bar, 15 μm. **e** Percentages of gonads with signal for S6K pThr389 antibody staining. The different colors represent the percentage of gonads with at 0, 1, 2, 3, or more than 3 cells stained in the premeiotic tip. Quantification was performed from gonads isolated from animals in the absence (−) or in the presence (+) of conditioned medium, and in the presence of metformin (M). The number of gonads analyzed is indicated on the top of the bars

To test the effect of modulating mTORC1 activity on sexual morph ratios, we treated mothers with pharmacological compounds. Mothers treated with rapamycin [63, 64] produced a greater proportion of F1 hermaphrodites than control mothers (Fig. 3a). mTORC1 signaling promotes nucleic acid synthesis, as long as nucleotide precursors are available [65]. Treatment with methotrexate, a chemical that suppresses the de novo purine synthesis enzymes [66], inhibits mTORC1 activity. We found that *A. freiburgensis* hermaphrodites treated with methotrexate generated mostly hermaphrodite offspring (Fig. 3a).

To investigate the kinase activity of mTORC1, we examined the expression of a well-characterized target protein, p70 S6K protein kinase (S6K) [58]. Antibody staining against the phosphorylated form of S6K (S6K pThr389) was detected in germline cells isolated from animals grown in low-density conditions (Fig. 3b–e). Most staining was associated with the chromatin, both in mitotic cells (Fig. 3b) and meiotic cells in late pachytene stages (Fig. 3c). Since AMPK and mTORC1 have opposing actions [67], we hypothesized that treatment of animals with metformin, an activator of AMPK, would inhibit mTORC1 signaling. Consistent with this hypothesis, we found that treatment of animals with metformin resulted in a smaller number of cells stained with SK6 pThr389 (Fig. 3e). Altogether, these results indicate that mTOR signaling is involved in intergenerational inheritance in *A. freiburgensis*.

### Insulin signaling is downregulated in animals in crowding conditions

**Fig. 4.**
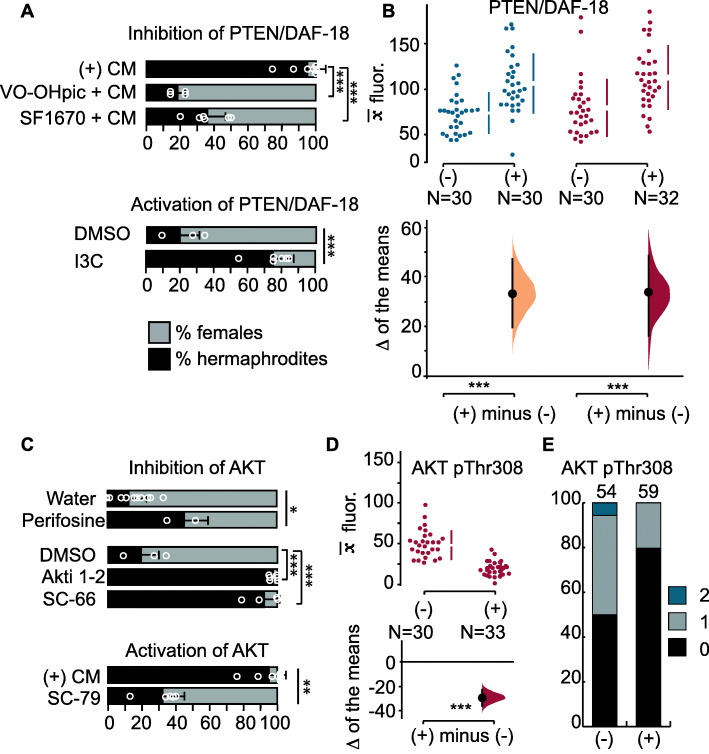
Regulation of PTEN/DAF-18 and AKT. **a** Mean percentage and SD of hermaphrodite and female F1 offspring from hermaphrodites treated with chemicals that activate or inhibit PTEN/DAF-18. (+) CM represents undiluted conditioned medium. The DMSO control and indole-3-carbinol (I3C) incubations were performed with diluted (1:10) CM. **b** Quantification of antibody staining with PTEN/DAF-18 in the maternal gonads. **c** Effect of pharmacological inhibition or activation of AKT on sexual morph ratios in the F1s. **d** Quantification of antibody staining for AKT pThr308 in the meiotic portion of the germline. **e** Quantification of premeiotic germline cells with staining with an antibody against AKT pThr308. Graphical representation in **b**, **d** as in Fig. 2b, and in **e** as in Fig. 3e. One-way analysis of variance (ANOVA) followed by a post hoc Holm-Sidak test for multiple comparisons (**a** inhibition of PTEN; **c** inhibition of AKT), Student’s *t* test (**a** activation of PTEN with I3C; **c** inhibition of AKT with perifosine), Mann-Whitney test (**c** activation of AKT with SC-79). *p ≤ 0.05, **p ≤ 0.01; ***p ≤ 0.001

The insulin signaling pathway regulates metabolism, development, and lifespan in a wide variety of animals. One of the regulators of the insulin pathway is a conserved phosphatase named PTEN (or DAF-18 in *C. elegans*) [68–70]. To test if PTEN/DAF-18 mediates the generation of hermaphrodites, we used the PTEN/DAF-18 inhibitors VO-OHpic [71] and SF1670 [72]. When in the presence of conditioned medium from high-density populations, hermaphrodites treated with those inhibitors generated mostly female offspring (Fig. 4a). Activation of PTEN/DAF-18 in hermaphrodites with the compound indole-3-carbinol under low population densities resulted in mostly hermaphrodites (Fig. 4a).

To examine the regulation of the insulin pathway in *A. freiburgensis*, we used an antibody against PTEN/DAF-18 to stain isolated gonads from hermaphrodites cultured in low- and high-density conditions. We found that the antibody against PTEN/DAF-18 stained more strongly the germline when hermaphrodites were incubated in high-density conditions than in low-density populations (Fig. 4b and Additional file 1: Figure S3).

One of the target proteins and effectors for insulin signaling is AKT kinase (also known as PKB) [73], which among several anabolic functions, also prevents chromatin condensation [74–76]. Maternal inhibition of AKT with the chemicals perifosine (prevents activation of AKT by affecting its subcellular localization) [77], Akti-1/2 (stabilizes the inactive conformation of AKT) [78], and SC-66 (allosteric inhibitor of AKT) [79] results in a higher proportion of hermaphrodite progeny (Fig. 4c). On the other hand, activation of AKT with SC-79 [80] prevents the generation of hermaphrodite progeny when the mother is in crowding conditions (Fig. 4c).

Maximal activation of AKT requires phosphorylation at residues Thr308 and Ser473 [81]. Immunostaining with antibodies against AKT pThr308 (Fig. 4d, e and Additional file 1: Figure S4) revealed that staining is prominently associated with the chromatin in germline cells of animals grown under non-crowding conditions. No such association is seen when animals are in crowding conditions (Fig. 4d). The same pattern is seen for AKT pSer473 (Additional file 1: Figure S4). Altogether, these results are consistent with the hypothesis that crowding conditions induce a lower insulin signaling, causing the production of hermaphrodite offspring.

### Changes in the maternal histone acetylation status correlate with changes in F1 development and mode of reproduction

**Fig. 5.**
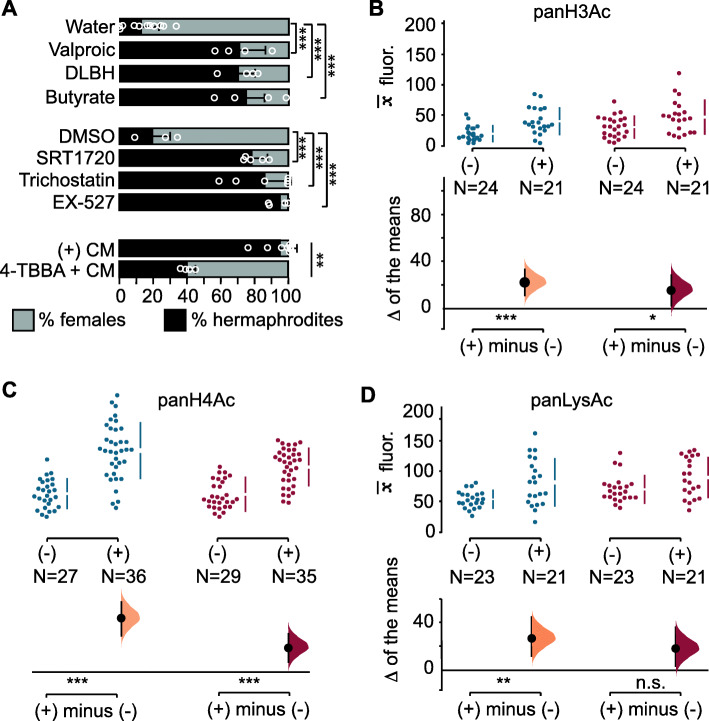
Regulation of acetylation levels. **a** Mean percentage and SD of hermaphrodite and female F1 offspring from hermaphrodites treated with chemicals. DLBH: DL-β hydroxybutyrate; TBBA: 4-tert-butylbenzoic acid. (1:10) CM was added to the medium, except plates with 4-TBBA, which had undiluted CM. Mean antibody fluorescence () for panH3Ac (**b**), panH4Ac (**c**), and panLysAc (**d**) in the premeiotic (blue) and meiotic portion (red) of the germline, in the absence (−) or presence (+) of conditioned medium. *N* = sample sizes. One-way analysis of variance (ANOVA) followed by a post hoc Holm-Sidak test for multiple comparisons (**a**), Mann-Whitney test (**a** 4-TBBA; **b–d**). n.s., *p* > 0.05; * = *p* ≤ 0.05; ** = *p* ≤ 0.01; *** = *p* ≤ 0.001

Energy-sensing pathways have been implicated in the regulation of acetylation of histones, histone modifiers, and cellular proteins [82]. To test if modulation of acetylation levels causes changes in sexual morph ratios, we induced hyperacetylation by treating *A. freiburgensis* hermaphrodites with the histone deacetylase inhibitors SRT1720 [83, 84], Trichostatin A [85], valproic acid [86, 87], D-β-hydroxybutyrate [88], sodium butyrate [89], and EX-527 [90]. In all cases, more hermaphrodites than females were produced relative to control (Fig. 5a). By contrast, incubating the mothers in high-density conditions together with the inhibitor of acetylation 4-tert-butylbenzoic acid [91] resulted in less hermaphrodite offspring (Fig. 5a).

To examine if acetylation patterns change in the germline when *A. freiburgensis* is in high population densities, we compared the level of antibody staining in gonads isolated from hermaphrodites cultured in the absence or presence of CM. Antibody staining against acetylated residues on histones 3 and 4 was at higher levels in the germline derived from animals cultured in the presence of CM compared to controls, both for premeiotic and meiotic portions (Fig. 5b,c and Additional file 1: Figure S5). The same trend was observed when using an antibody that binds to all acetylated proteins (panLysAc), although differences were detected only for the premeiotic portion of the germline (Fig. 5d).

## Discussion

*Auanema* nematodes have been isolated from similar environments as *C. elegans* [92], which consists of ephemeral habitats with microbe-rich organic decomposing matter [23, 93]. Due to rapid population growth and quick depletion of resources, the ecology of these nematodes is characterized by a boom-bust population dynamics. In contrast to *C. elegans*, the developmental and phenotypic response to stress in *A. freiburgensis* occurs across a generation instead of within the same generation: maternal sensing of pheromones secreted by conspecifics induces the production of stress- and starvation-resistant dauer larvae. This indicates that the *A. freiburgensis* mother can predict the environmental conditions to which the offspring is likely to be exposed and adjusts the F1 phenotype (dauer larvae) to temporarily survive in the absence of food. The *Auanema* dauers have migratory behaviors and always develop into selfing hermaphrodites [23]. By producing dauers that develop into hermaphrodites, a new population can be established even when the colonizing event is mediated by a single individual [94]. This type of intergenerational inheritance, in which parental effects increase the fitness of both offspring and parents, has hallmarks for being adaptive [95].

Here we show that activators of AMPK and insulin signaling activators or mTORC1 inhibitors intergenerationally induce the formation of dauers and hermaphrodites in *A. freiburgensis*. These results indicate that highly conserved energy-sensing pathways are involved in mediating intergenerational inheritance in *A. freiburgensis* to generate stress-resistant offspring. Consistent with the role of these pathways in dauer formation in *A. freiburgensis*, *C. elegans* integrates nutrient sensing using the same abovementioned signaling pathways to regulate developmental progression through larval stages [96]. How exactly do these energy-sensing pathways regulate phenotypic plasticity in the *A. freiburgensis* F1s? One possible mechanism is the direct regulation of the chromatin status in the maternal germline by the energy-sensing enzymes. Thus, activation of transcription of specific genes in the germline may determine the phenotype of the following generation. AMPK, for instance, has been shown to phosphorylate histones, which results in the activation of transcription [97]. Consistent with this, we found that protein levels increase for the activated form of AMPK when *A. freiburgensis* is under crowding conditions and is detected in close association with the chromatin of germline cells. By phosphorylating histones, AMPK has been shown to facilitate histone acetylation [98], thus promoting the transcription of a new set of genes [99]. However, AMPK also regulates cytoplasmic proteins responsible for the metabolic status of the cell and interacts with the mTOR, sirtuin, and insulin pathways [100]. Future research will reveal whether the roles of these pathways for intergenerational inheritance are more important in the cytosolic or nuclear context.

AMPK may also indirectly influence the chromatin status via activation of histone acetyltransferases (HATs) or inactivation of histone deacetyltransferases (HDAC), as demonstrated for other model systems [101, 102]. In *A. freiburgensis*, higher acetylation levels in the chromatin of the germline induced by crowding conditions result in stress-resistant offspring (Fig. 5). It remains to be established whether these acetylation levels are the result of direct phosphorylation of HATs and HDACs by AMPK, or indirectly by natural metabolites. As we show in Fig. 5a, natural metabolites indicative of metabolic stress that inhibit deacetyltransferases, such as D-β-hydroxybutyrate and butyrate [101], induce the production of stress-resistant offspring.

HDACs are subdivided into two protein subfamilies, which differ in protein structure and mechanism of action: the classical HDAC family and the sirtuins family [103]. The pharmacological compounds SRT1720 and EX-527 were designed to inhibit sirtuins [84, 90], whereas sodium butyrate [104], Trichostatin A [85], valproic acid [105], and D-β-hydroxybutyrate [101] inhibit the classic HDACs. Given that the same intergenerational effects were obtained with compounds that interfere with different subfamilies of HDACs, which may also have different target proteins, these results would suggest that the effects are not direct. Therefore, it is still unclear if the intergenerational effects are due to changes in the chromatin acetylation or in other subcellular components.

The strongest responses to the pharmacological compounds for the production of hermaphrodite progeny occurred when the animals were concomitantly exposed to diluted CM. In the complete absence of CM, only a few compounds elicited a strong response. This may indicate that pheromones in the CM activate more than one pathway and that they have to act in combination to elicit the full effect. Our findings that several energy-sensing pathways are involved in this process in *A. freiburgensis*, and that AMPK, insulin, and TOR pathways are cross-regulated, are indicative of this hypothesis [106–108].

The concentration of the compounds used in our studies and in *C. elegans* are relatively high compared to the ones used in mammalian cells [109–111]. This is because nematodes have several physical and physiological adaptations that counteract xenobiotic agents [112]. Like all pharmacological approaches, interpretation of the results must take into consideration possible lack of specificity [48, 113–115]. To ameliorate the possibility of lack of specificity for AMPK activation, for instance, we used compounds that act through several mechanisms (high production of AMP, allosteric binding, protection against dephosphorylation, activation of phosphorylation). Genetic approaches using loss- and gain-of-function mutants will help to address some of the abovementioned concerns [116].

As far as we know, the association of activated AMPK and S6K with the chromatin of germline cells has not been established in other organisms. The presence of AKT in the nucleus of germline cells may be associated with chromatin condensation, which would be reflected in transcription rates [74].

## Conclusions

Our results suggest that these energy-sensing effectors acquired a new role in intergenerational inheritance in *A. freiburgensis* to regulate gene expression that influences the phenotype of subsequent generations. Given that AMPK, TOR, and insulin pathways are highly conserved in evolution, it is possible that they also mediate nongenetic inheritance via the germline in other organisms in which diet plays a role in determining phenotypic plasticity. Although the epidemiological studies in humans are indicative of diet playing such a role, the mechanisms for this are unknown [117]. The findings in this study provide the basis to test such a hypothesis.

## Methods

### Strain and culture

We used the *Caenorhabditis elegans* N2 strain, and the *Auanema freiburgensis* strains SB372 [23] and JU1782. The *A. freiburgensis* JU1782 strain was isolated from rotting *Petasites* stems sampled in Ivry, Val-de-Marne, France, in September 2009 by Marie-Anne Félix. Nematodes were cultured at 20 °C on standard Nematode Growth Medium (NGM) [118] plates seeded with *Escherichia coli* OP50-1 strain. NGM medium was supplemented with 25 μg/mL nystatin and 50 μg/mL streptomycin to prevent microbial contamination.

### Sexing of progeny

To synchronize the age of the mothers, we collected dauers. *A. freiburgensis* dauers develop into hermaphrodite adults within 24 h at 20 °C [23]. Dauer larvae are easily identified by their darker intestine and thinner body compared to similar-sized L3 larvae (which develop into females). Each dauer larva was placed on a 6-cm seeded NGM plate and incubated at 20 °C to develop into adulthood. Each egg laid by the parental (P0) generation was placed into single wells of a 96-well microtiter plate. After 3–5 days, the F1 was scored for their sexual morphology: hermaphrodites were identified by their ability to produce offspring in the absence of a mating partner, females by the lack of progeny, and males by their blunt tails [23]. We calculated sexual morph percentages based only on non-male progeny (hermaphrodites or females). This is because males are not determined by environmental cues, but by sex chromosome number. Raw data used to calculate sexual morph percentages are in the data repository [119].

### Diet restriction experiments

To test the effect of starving the parents in the sexual morph ratios of *A. freiburgensis* hermaphrodites, we followed the diet restriction protocol previously described [120]. Briefly, dauers were collected to plates with *E. coli* OP50 and allowed to mature to adulthood at 20 °C for 24 h. They were then transferred to 6-well tissue culture plates, with each well containing 5 ml of standard NGM medium supplemented with 1 mg/ml erythromycin. Hermaphrodites were left laying eggs in the absence of bacterial food for 2–3 days. F1 eggs or larvae were collected to be sexed as described above.

### Assay with conditioned medium and treatment with pharmacological chemical compounds

To induce *A. freiburgensis* hermaphrodite offspring, the parent hermaphrodites (P0 generation) were incubated in the presence of conditioned medium (CM) at 20 °C [121]. The CM was derived from 2- to 3-week-old *A. freiburgensis* liquid cultures (M9 medium with *E. coli* OP50-1). Each P0 was placed at the L4 stage onto a 6-cm plate containing NGM and CM. To simulate high-density conditions, 50 mg of lyophilized CM was dissolved in 200 μl of an overnight culture of OP50-1 and spotted onto the plate. F1 eggs were collected for 3–4 days. Each egg was transferred into a single well of a 48-well microtiter plate containing NGM and OP50-1, but no conditioned medium.

For the pharmacological manipulation of signaling pathways, we added compounds to the NGM and OP50-1. The concentration of the compounds was calculated for the volume of the NGM and OP50-1 used. P0 hermaphrodites were incubated with the compounds for 48–96 h at 20 °C. Information about the providers and catalog number for the compounds used in this study are listed in the data repository figshare [119]. Alignment of the protein sequences of the immunogens used to raise the antibodies and their predicted *A. freiburgensis* orthologs are in Additional file 1: Figure S6.

Chemical compounds were used at the following concentrations: 100 mM metformin, 6 mM phenformin, 1 μM rotenone, 5 μM forskolin, 30 μM fluvastatin, 0.5 mM AICAR, 0.5 mM 8-Br-cAMP, 5 mM salicylate, 10 μM ZLN204, 30 μM O-304, 1 μM dorsomorphin, 100 μM rapamycin, 100 μM methotrexate, 100 nM VO-OHpic, 20 μM indole-3-carbinol, 10 μM SRT1720, 100 μM trichostatin A, 4 mM valproic acid, 5 mM DL-beta-hydroxybutyrate, 5 mM sodium butyrate, 100 μM EX-527, 3 mM 4-tert-butylbenzoic acid, 75 nM SC-66, 300 nM Akti-1/2, 20 μM perifosine, and 10 nM SC-79. For nematodes incubated with diluted CM, we used 5 mg of freeze-dried CM dissolved in 200 μl *E. coli* OP50-1.

### Immunohistochemistry

To ascertain that antibodies would cross-react with *A. freiburgensis* tissues, tblastn searches [122] were performed using the *A. freiburgensis* transcriptome (details to be published elsewhere). Hermaphrodites were dissected on a slide (Superfrost microscope slide, VWR) in PBS 1× buffer. Dissected gonads were covered by a coverslip and placed on a frozen metal block at − 20 °C for at least 10 min, and fixed for 2 min in a 95% methanol solution at − 20 °C. This was followed by 30 min in a fixative solution [PBS 1×, 80 mM HEPES (pH = 7.0–7.4), 1.6 mM MgSO4, 0.8 mM EDTA (pH = 8.0), 4% paraformaldehyde] in a humid chamber at room temperature. Slides were washed twice with PBST (PBS + 0.1% Triton X-100) for 5 min and blocked in PBST + 0.5% BSA for 45–60 min. The source of primary and secondary antibodies, as well as dilutions used, is listed in figshare [119]. All antibodies were diluted in PBST. Incubation with the primary antibodies was performed at 4 °C overnight. Slides were then washed twice in PBST for 10 min each, and the corresponding secondary antibody was added and incubated for 2 h at room temperature. Slides were washed in PBST as above to remove the excess of the secondary antibody and then one drop of Fluoroshield Mounting Medium with 4′,6-diamidino-2-phenylindole (DAPI) (Abcam, #ab104139) was added on the immunostained samples.

Images were taken with a × 60 objective in 2.40 μm z-stack intervals (12 sections) with a DeltaVision microscope (Olympus). Acquisition and constrained iterative deconvolution of the images from DeltaVision were processed using the softWoRx software (Applied Precision). The deconvolved images were processed using restoration methods (instead of deblurring methods) [123]. Restoration methods do not affect the quantitative nature of the final restored product because linearity is better preserved [124]. The intensity of fluorescence for the secondary antibodies was measured in images in the TIFF format, using the ImageJ software (NIH Image, Bethesda, MD). Based on the morphology of the nuclei in DAPI-stained cells, the germline was subdivided into two sections: the premeiotic tip (also known as mitotic region) and the meiotic region. As observed in *C. elegans* [125], cell nuclei between the mitotic and the meiotic region of the *A. freiburgensis* germline have a characteristic crescent-shaped morphology. This region is known as the “transition zone,” with cells in the leptotene and zygotene of the meiotic cycle. For normalization and calculation of fluorescence, we estimated fluorescent signals using drawing tools in ImageJ software and defined “set measurements” (including areas, integrated intensity, and mean grey value) and then selected “Measure” from the Analyze menu.

### Western blot

Protein extraction and buffer preparation were performed following the protocol of [126]. Six hundred adult hermaphrodites were collected for each sample: control (OP50-1 only) and experimental (50 mg conditioned medium powder per 200 μl of OP50-1) samples. Protein concentration was measured using Bradford assay (Bradford Reagent, Bio-Rad). We loaded approximately 100 μg of protein. The primary antibodies, against Phospho-AMPKα (Thr172) and PAR-4/LKB1, were used at 1:1000 dilution. The source of primary and secondary antibodies, as well as dilutions used for them, is listed in figshare [119]. To detect the signal for the antibodies, we used the Amersham™ ECL™ Western Blotting Detection Reagents (RPN2209). For *A. freiburgensis* gene_ID, immunogen UniProtKB_ID and percentage identity of the alignment, see in figshare [119]. Uncropped images for Western blots are in Additional file 1: Figure S7.

### Statistical analyses

Results were presented using the most recent developments in data analysis and presentation [127], showing the raw data as “bee swarm” plots. They summarize the data showing the mean and the 95% confidence interval (CI), as well as the sampling error distribution diagrammed as a filled curve. These plots provide transparency of the comparison being made, visual clarity, and statistical evaluation of the data. For the effect of pharmacological compounds on sex ratios, Student’s *t* test, one-way analysis of variance (ANOVA) followed by the Holm-Sidak method for multiple comparisons or Kruskal-Wallis one-way analysis of variance on ranks followed by Dunn’s method were performed. We used the software package SigmaPlot 12.0 (Systat Software, Inc., San Jose, CA, USA). Details of sample sizes, statistical tests and *p* values are in the data repository figshare [119].

## Supplementary Information

**Additional file 1: Figure S1**. STRAD and LKB1 antibody staining are higher in animals in crowding conditions. **Figure S2**. Metformin and trichostatin affect the F1 sex ratios in the absence of diluted CM. **Figure S3**. PTEN/DAF-18 in the germline is cytoplasmic and higher in non-crowding conditions. **Figure S4**. AKT pThr308 in the germline is nuclear and higher in crowding conditions. **Figure S5**. Acetylation in the germline is nuclear and higher in crowding conditions. **Figure S6**. Alignment of the protein sequences of the immunogens used to raise the antibodies and their predicted *A. freiburgensis* orthologs. **Figure S7**. Uncropped images of Western blots.

## Data Availability

Correspondence and requests should be addressed to A.P.-d.S. All data generated or analyzed during this study are included in this published article and its supplementary information files.

## References

[1] Lempradl A (2020). Germ cell-mediated mechanisms of epigenetic inheritance. Semin Cell Dev Biol.

[2] Wang Y, Liu H, Sun Z (2017). Lamarck rises from his grave: parental environment-induced epigenetic inheritance in model organisms and humans. Biol Rev Camb Philos Soc.

[3] Willis AR, Sukhdeo R, Reinke AW. Remembering your enemies: mechanisms of within-generation and multigenerational immune priming in Caenorhabditis elegans. Febs j. 2020. 10.1111/febs.15509 32767821

[4] Burton NO, Riccio C, Dallaire A, Price J, Jenkins B, Koulman A, Miska EA (2020). Cysteine synthases CYSL-1 and CYSL-2 mediate *C. elegans* heritable adaptation to *P. vranovensis* infection. Nat Commun.

[5] Posner R, Toker IA, Antonova O, Star E, Anava S, Azmon E, Hendricks M, Bracha S, Gingold H, Rechavi O (2019). Neuronal small RNAs control behavior transgenerationally. Cell.

[6] Kaletsky R, Moore RS, Vrla GD, Parsons LR, Gitai Z, Murphy CT. C. elegans interprets bacterial non-coding RNAs to learn pathogenic avoidance. Nature. 2020;586(7829):445–51. 10.1038/s41586-020-2699-5 PMC8547118 32908307

[7] Webster AK, Jordan JM, Hibshman JD, Chitrakar R, Baugh LR (2018). Transgenerational effects of extended dauer diapause on starvation survival and gene expression plasticity in Caenorhabditis elegans. Genetics.

[8] Burton NO, Furuta T, Webster AK, Kaplan REW, Baugh LR, Arur S, Horvitz HR. Insulin-like signalling to the maternal germline controls progeny response to osmotic stress.Nat Cell Biol. 2017;19:252. 10.1038/ncb3470 PMC5332277 28166192

[9] Perez MF, Lehner B (2019). Intergenerational and transgenerational epigenetic inheritance in animals. Nat Cell Biol.

[10] Jablonka E (2013). Epigenetic inheritance and plasticity: the responsive germline. Prog Biophys Mol Biol.

[11] Öst A, Lempradl A, Casas E, Weigert M, Tiko T, Deniz M, Pantano L, Boenisch U, Itskov Pavel M, Stoeckius M (2014). Paternal diet defines offspring chromatin state and intergenerational obesity. Cell.

[12] Chen Q, Yan M, Cao Z, Li X, Zhang Y, Shi J, Feng GH, Peng H, Zhang X, Zhang Y, Qian J, Duan E, Zhai Q, Zhou Q (2016). Sperm tsRNAs contribute to intergenerational inheritance of an acquired metabolic disorder. Science.

[13] Sharma U, Conine CC, Shea JM, Boskovic A, Derr AG, Bing XY, Belleannee C, Kucukural A, Serra RW, Sun F, Song L, Carone BR, Ricci EP, Li XZ, Fauquier L, Moore MJ, Sullivan R, Mello CC, Garber M, Rando OJ (2016). Biogenesis and function of tRNA fragments during sperm maturation and fertilization in mammals. Science.

[14] Perez MF, Francesconi M, Hidalgo-Carcedo C, Lehner B (2017). Maternal age generates phenotypic variation in Caenorhabditis elegans. Nature.

[15] Jordan JM, Hibshman JD, Webster AK, Kaplan REW, Leinroth A, Guzman R, Maxwell CS, Chitrakar R, Bowman EA, Fry AL, Hubbard EJA, Baugh LR (2019). Insulin/IGF signaling and vitellogenin provisioning mediate intergenerational adaptation to nutrient stress. Curr Biol.

[16] Mousseau TA, Fox CW (1998). The adaptive significance of maternal effects. Trends Ecol Evol.

[17] Baugh LR, Day T (2020). Nongenetic inheritance and multigenerational plasticity in the nematode C. elegans. eLlife.

[18] Agrawal AA, Laforsch C, Tollrian R (1999). Transgenerational induction of defences in animals and plants. Nature.

[19] Greer EL, Maures TJ, Ucar D, Hauswirth AG, Mancini E, Lim JP, Benayoun BA, Shi Y, Brunet A (2011). Transgenerational epigenetic inheritance of longevity in *Caenorhabditis elegans*. Nature.

[20] Rechavi O, Houri-Ze'evi L, Anava S, Goh WS, Kerk SY, Hannon GJ, Hobert O (2014). Starvation-induced transgenerational inheritance of small RNAs in *C. elegans*. Cell.

[21] Félix MA (2004). Alternative morphs and plasticity of vulval development in a rhabditid nematode species. Dev Genes Evol.

[22] Chaudhuri J, Bose N, Tandonnet S, Adams S, Zuco G, Kache V, Parihar M, von Reuss SH, Schroeder FC, Pires-daSilva A (2015). Mating dynamics in a nematode with three sexes and its evolutionary implications. Sci Rep.

[23] Kanzaki N, Kiontke K, Tanaka R, Hirooka Y, Schwarz A, Muller-Reichert T, Chaudhuri J, Pires-daSilva A (2017). Description of two three-gendered nematode species in the new genus *Auanema* (Rhabditina) that are models for reproductive mode evolution. Sci Rep.

[24] Shakes DC, Neva BJ, Huynh H, Chaudhuri J, Pires-daSilva A (2011). Asymmetric spermatocyte division as a mechanism for controlling sex ratios. Nat Commun.

[25] Chaudhuri J, Kache V, Pires-daSilva A (2011). Regulation of sexual plasticity in a nematode that produces males, females, and hermaphrodites. Curr Biol.

[26] Johnigk SA, Ehlers RU (1999). Juvenile development and life cycle of Heterorhabditis bacteriophora and H-indica (Nematoda : Heterorhabditidae). Nematology.

[27] Cassada RC, Russell RL (1975). The dauerlarva, a post-embryonic developmental variant of the nematode *Caenorhabditis elegans*. Dev Biol.

[28] Demoinet E, Li S, Roy R (2017). AMPK blocks starvation-inducible transgenerational defects in Caenorhabditis elegans. Proc Natl Acad Sci U S A.

[29] Jobson MA, Jordan JM, Sandrof MA, Hibshman JD, Lennox AL, Baugh LR. Transgenerational effects of early life starvation on growth, reproduction and stress resistance in *Caenorhabditis elegans*. Genetics. 2015. 10.1534/genetics.115.178699 PMC4566263 26187123

[30] Apfeld J, O'Connor G, McDonagh T, DiStefano PS, Curtis R. The AMP-activated protein kinase AAK-2 links energy levels and insulin-like signals to lifespan in *C. elegans*. Genes Dev. 2004;18(24):3004–9. 10.1101/gad.1255404 PMC535911 15574588

[31] Hardie DG, Ross FA, Hawley SA (2012). AMPK: a nutrient and energy sensor that maintains energy homeostasis. Nat Rev Mol Cell Biol.

[32] Carling D (2004). The AMP-activated protein kinase cascade--a unifying system for energy control. Trends Biochem Sci.

[33] Narbonne P, Roy R (2006). Inhibition of germline proliferation during C. elegans dauer development requires PTEN, LKB1 and AMPK signalling. Development.

[34] Fukuyama M, Sakuma K, Park R, Kasuga H, Nagaya R, Atsumi Y, Shimomura Y, Takahashi S, Kajiho H, Rougvie A, Kontani K, Katada T (2012). C. elegans AMPKs promote survival and arrest germline development during nutrient stress. Biol Open.

[35] Stein SC, Woods A, Jones NA, Davison MD, Carling D (2000). The regulation of AMP-activated protein kinase by phosphorylation. Biochem J.

[36] Lee H, Cho JS, Lambacher N, Lee J, Lee SJ, Lee TH, Gartner A, Koo HS (2008). The Caenorhabditis elegans AMP-activated protein kinase AAK-2 is phosphorylated by LKB1 and is required for resistance to oxidative stress and for normal motility and foraging behavior. J Biol Chem.

[37] Steinberg GR, Carling D (2019). AMP-activated protein kinase: the current landscape for drug development. Nat Rev Drug Discov.

[38] El-Mir MY, Nogueira V, Fontaine E, Averet N, Rigoulet M, Leverve X (2000). Dimethylbiguanide inhibits cell respiration via an indirect effect targeted on the respiratory chain complex I. J Biol Chem.

[39] Owen MR, Doran E, Halestrap AP (2000). Evidence that metformin exerts its anti-diabetic effects through inhibition of complex 1 of the mitochondrial respiratory chain. Biochem J.

[40] Zhou G, Myers R, Li Y, Chen Y, Shen X, Fenyk-Melody J, Wu M, Ventre J, Doebber T, Fujii N, Musi N, Hirshman MF, Goodyear LJ, Moller DE (2001). Role of AMP-activated protein kinase in mechanism of metformin action. J Clin Invest.

[41] Sakamoto K, Goransson O, Hardie DG, Alessi DR (2004). Activity of LKB1 and AMPK-related kinases in skeletal muscle: effects of contraction, phenformin, and AICAR. Am J Physiol Endocrinol Metab.

[42] Shaw RJ, Kosmatka M, Bardeesy N, Hurley RL, Witters LA, DePinho RA, Cantley LC (2004). The tumor suppressor LKB1 kinase directly activates AMP-activated kinase and regulates apoptosis in response to energy stress. Proc Natl Acad Sci U S A.

[43] Huang X, Wullschleger S, Shpiro N, McGuire VA, Sakamoto K, Woods YL, McBurnie W, Fleming S, Alessi DR (2008). Important role of the LKB1-AMPK pathway in suppressing tumorigenesis in PTEN-deficient mice. Biochem J.

[44] Toyama EQ, Herzig S, Courchet J, Lewis TL, Loson OC, Hellberg K, Young NP, Chen H, Polleux F, Chan DC (2016). Metabolism. AMP-activated protein kinase mediates mitochondrial fission in response to energy stress. Science.

[45] Hou WL, Yin J, Alimujiang M, Yu XY, Ai LG, Bao YQ, Liu F, Jia WP (2018). Inhibition of mitochondrial complex I improves glucose metabolism independently of AMPK activation. J Cell Mol Med.

[46] Seamon KB, Daly JW, Metzger H, de Souza NJ, Reden J (1983). Structure-activity relationships for activation of adenylate cyclase by the diterpene forskolin and its derivatives. J Med Chem.

[47] Xenos ES, Stevens SL, Freeman MB, Cassada DC, Goldman MH (2005). Nitric oxide mediates the effect of fluvastatin on intercellular adhesion molecule-1 and platelet endothelial cell adhesion molecule-1 expression on human endothelial cells. Ann Vasc Surg.

[48] Corton JM, Gillespie JG, Hawley SA, Hardie DG (1995). 5-aminoimidazole-4-carboxamide ribonucleoside. A specific method for activating AMP-activated protein kinase in intact cells?. Eur J Biochem.

[49] Hussey R, Stieglitz J, Mesgarzadeh J, Locke TT, Zhang YK, Schroeder FC, Srinivasan S (2017). Pheromone-sensing neurons regulate peripheral lipid metabolism in Caenorhabditis elegans. Plos Genet.

[50] Hawley SA, Fullerton MD, Ross FA, Schertzer JD, Chevtzoff C, Walker KJ, Peggie MW, Zibrova D, Green KA, Mustard KJ, Kemp BE, Sakamoto K, Steinberg GR, Hardie DG (2012). The ancient drug salicylate directly activates AMP-activated protein kinase. Science.

[51] Zhang LN, Xu L, Zhou HY, Wu LY, Li YY, Pang T, Xia CM, Qiu BY, Gu M, Dong TC, Li JY, Shen JK, Li J (2013). Novel small-molecule AMP-activated protein kinase allosteric activator with beneficial effects in db/db mice. Plos One.

[52] Steneberg P, Lindahl E, Dahl U, Lidh E, Straseviciene J, Backlund F, Kjellkvist E, Berggren E, Lundberg I, Bergqvist I, Ericsson M, Eriksson B, Linde K, Westman J, Edlund T, Edlund H (2018). PAN-AMPK activator O304 improves glucose homeostasis and microvascular perfusion in mice and type 2 diabetes patients. JCI Insight.

[53] Watts JL, Morton DG, Bestman J, Kemphues KJ (2000). The C. elegans par-4 gene encodes a putative serine-threonine kinase required for establishing embryonic asymmetry. Development.

[54] Woods A, Johnstone SR, Dickerson K, Leiper FC, Fryer LG, Neumann D, Schlattner U, Wallimann T, Carlson M, Carling D (2003). LKB1 is the upstream kinase in the AMP-activated protein kinase cascade. Curr Biol.

[55] Hawley SA, Boudeau J, Reid JL, Mustard KJ, Udd L, Makela TP, Alessi DR, Hardie DG (2003). Complexes between the LKB1 tumor suppressor, STRAD alpha/beta and MO25 alpha/beta are upstream kinases in the AMP-activated protein kinase cascade. J Biol.

[56] Baas AF, Boudeau J, Sapkota GP, Smit L, Medema R, Morrice NA, Alessi DR, Clevers HC (2003). Activation of the tumour suppressor kinase LKB1 by the STE20-like pseudokinase STRAD. EMBO J.

[57] Boudeau J, Baas AF, Deak M, Morrice NA, Kieloch A, Schutkowski M, Prescott AR, Clevers HC, Alessi DR (2003). MO25alpha/beta interact with STRADalpha/beta enhancing their ability to bind, activate and localize LKB1 in the cytoplasm. EMBO J.

[58] Kapahi P, Chen D, Rogers AN, Katewa SD, Li PW, Thomas EL, Kockel L (2010). With TOR, less is more: a key role for the conserved nutrient-sensing TOR pathway in aging. Cell Metab.

[59] Ma XM, Blenis J (2009). Molecular mechanisms of mTOR-mediated translational control. Nat Rev Mol Cell Biol.

[60] Wullschleger S, Loewith R, Hall MN (2006). TOR signaling in growth and metabolism. Cell.

[61] Zoncu R, Efeyan A, Sabatini DM (2011). mTOR: from growth signal integration to cancer, diabetes and ageing. Nat Rev Mol Cell Biol.

[62] Laplante M, Sabatini DM (2012). mTOR signaling in growth control and disease. Cell.

[63] Heitman J, Movva NR, Hall MN (1991). Targets for cell cycle arrest by the immunosuppressant rapamycin in yeast. Science.

[64] Robida-Stubbs S, Glover-Cutter K, Lamming DW, Mizunuma M, Narasimhan SD, Neumann-Haefelin E, Sabatini DM, Blackwell TK (2012). TOR signaling and rapamycin influence longevity by regulating SKN-1/Nrf and DAF-16/FoxO. Cell Metab.

[65] Hoxhaj G, Hughes-Hallett J, Timson RC, Ilagan E, Yuan M, Asara JM, Ben-Sahra I, Manning BD (2017). The mTORC1 signaling network senses changes in cellular purine nucleotide levels. Cell Rep.

[66] Rajagopalan PT, Zhang Z, McCourt L, Dwyer M, Benkovic SJ, Hammes GG (2002). Interaction of dihydrofolate reductase with methotrexate: ensemble and single-molecule kinetics. Proc Natl Acad Sci U S A.

[67] Hindupur SK, Gonzalez A, Hall MN (2015). The opposing actions of target of rapamycin and AMP-activated protein kinase in cell growth control. Cold Spring Harb Perspect Biol.

[68] Steck PA, Pershouse MA, Jasser SA, Yung WK, Lin H, Ligon AH, Langford LA, Baumgard ML, Hattier T, Davis T (1997). Identification of a candidate tumour suppressor gene, MMAC1, at chromosome 10q23.3 that is mutated in multiple advanced cancers. Nat Genet.

[69] Ogg S, Ruvkun G (1998). The C. elegans PTEN homolog, DAF-18, acts in the insulin receptor-like metabolic signaling pathway. Mol Cell.

[70] Solari F, Bourbon-Piffaut A, Masse I, Payrastre B, Chan AM, Billaud M (2005). The human tumour suppressor PTEN regulates longevity and dauer formation in Caenorhabditis elegans. Oncogene.

[71] Rosivatz E, Matthews JG, McDonald NQ, Mulet X, Ho KK, Lossi N, Schmid AC, Mirabelli M, Pomeranz KM, Erneux C (2006). A small molecule inhibitor for phosphatase and tensin homologue deleted on chromosome 10 (PTEN). ACS Chem Biol.

[72] Li Y, Prasad A, Jia Y, Roy SG, Loison F, Mondal S, Kocjan P, Silberstein LE, Ding S, Luo HR (2011). Pretreatment with phosphatase and tensin homolog deleted on chromosome 10 (PTEN) inhibitor SF1670 augments the efficacy of granulocyte transfusion in a clinically relevant mouse model. Blood.

[73] Paradis S, Ruvkun G (1998). Caenorhabditis elegans Akt/PKB transduces insulin receptor-like signals from AGE-1 PI3 kinase to the DAF-16 transcription factor. Genes Dev.

[74] Martelli AM, Tabellini G, Bressanin D, Ognibene A, Goto K, Cocco L, Evangelisti C (2012). The emerging multiple roles of nuclear Akt. Biochim Biophys Acta Mol Cell Res.

[75] Manning BD, Cantley LC (2007). AKT/PKB signaling: navigating downstream. Cell.

[76] Manning BD, Toker A (2017). AKT/PKB Signaling: navigating the network. Cell.

[77] Kondapaka SB, Singh SS, Dasmahapatra GP, Sausville EA, Roy KK (2003). Perifosine, a novel alkylphospholipid, inhibits protein kinase B activation. Mol Cancer Ther.

[78] Barnett SF, Defeo-Jones D, Fu S, Hancock PJ, Haskell KM, Jones RE, Kahana JA, Kral AM, Leander K, Lee LL (2005). Identification and characterization of pleckstrin-homology-domain-dependent and isoenzyme-specific Akt inhibitors. Biochem J.

[79] Jo H, Lo PK, Li Y, Loison F, Green S, Wang J, Silberstein LE, Ye K, Chen H, Luo HR (2011). Deactivation of Akt by a small molecule inhibitor targeting pleckstrin homology domain and facilitating Akt ubiquitination. Proc Natl Acad Sci U S A.

[80] Jo H, Mondal S, Tan D, Nagata E, Takizawa S, Sharma AK, Hou Q, Shanmugasundaram K, Prasad A, Tung JK, Tejeda AO, Man H, Rigby AC, Luo HR (2012). Small molecule-induced cytosolic activation of protein kinase Akt rescues ischemia-elicited neuronal death. Proc Natl Acad Sci U S A.

[81] Alessi DR, Andjelkovic M, Caudwell B, Cron P, Morrice N, Cohen P, Hemmings BA (1996). Mechanism of activation of protein kinase B by insulin and IGF-1. EMBO J.

[82] Salminen A, Kauppinen A, Kaarniranta K (2016). AMPK/Snf1 signaling regulates histone acetylation: Impact on gene expression and epigenetic functions. Cel Signal.

[83] Zarse K, Schmeisser S, Birringer M, Falk E, Schmoll D, Ristow M (2010). Differential effects of resveratrol and SRT1720 on lifespan of adult Caenorhabditis elegans. Horm Metab Res.

[84] Milne JC, Lambert PD, Schenk S, Carney DP, Smith JJ, Gagne DJ, Jin L, Boss O, Perni RB, Vu CB, Bemis JE, Xie R, Disch JS, Ng PY, Nunes JJ, Lynch AV, Yang H, Galonek H, Israelian K, Choy W, Iffland A, Lavu S, Medvedik O, Sinclair DA, Olefsky JM, Jirousek MR, Elliott PJ, Westphal CH (2007). Small molecule activators of SIRT1 as therapeutics for the treatment of type 2 diabetes. Nature.

[85] Yoshida M, Kijima M, Akita M, Beppu T (1990). Potent and specific inhibition of mammalian histone deacetylase both in vivo and in vitro by trichostatin A. J Biol Chem.

[86] Evason K, Collins JJ, Huang C, Hughes S, Kornfeld K (2008). Valproic acid extends Caenorhabditis elegans lifespan. Aging Cell.

[87] Forthun RB, Sengupta T, Skjeldam HK, Lindvall JM, McCormack E, Gjertsen BT, Nilsen H (2012). Cross-species functional genomic analysis identifies resistance genes of the histone deacetylase inhibitor valproic acid. Plos One.

[88] Edwards C, Canfield J, Copes N, Rehan M, Lipps D, Bradshaw PC (2014). D-beta-hydroxybutyrate extends lifespan in *C. elegans*. Aging (Albany NY).

[89] Zhang M, Poplawski M, Yen K, Cheng H, Bloss E, Zhu X, Patel H, Mobbs CV (2009). Role of CBP and SATB-1 in aging, dietary restriction, and insulin-like signaling. Plos Biol.

[90] Solomon JM, Pasupuleti R, Xu L, McDonagh T, Curtis R, DiStefano PS, Huber LJ (2006). Inhibition of SIRT1 catalytic activity increases p53 acetylation but does not alter cell survival following DNA damage. Mol Cell Biol.

[91] Chen YP, Catbagan CC, Bowler JT, Gokey T, Goodwin ND, Guliaev AB, Wu W, Amagata T (2014). Evaluation of benzoic acid derivatives as sirtuin inhibitors. Bioorg Med Chem Lett.

[92] Félix MA, Duveau F (2012). Population dynamics and habitat sharing of natural populations of *Caenorhabditis elegans* and *C. briggsae*. BMC Biol.

[93] Schulenburg H, Felix MA (2017). The natural biotic environment of *Caenorhabditis elegans*. Genetics.

[94] Baker HG (1955). Self-compatibility and establishment after “long distance” dispersal. Evolution.

[95] Uller T (2008). Developmental plasticity and the evolution of parental effects. Trends Ecol Evol.

[96] Rashid S, Pho KB, Mesbahi H, MacNeil LT (2020). Nutrient sensing and response drive developmental progression in Caenorhabditis elegans. Bioessays.

[97] Bungard D, Fuerth BJ, Zeng PY, Faubert B, Maas NL, Viollet B, Carling D, Thompson CB, Jones RG, Berger SL (2010). Signaling kinase AMPK activates stress-promoted transcription via histone H2B phosphorylation. Science.

[98] Lo WS, Duggan L, Emre NC, Belotserkovskya R, Lane WS, Shiekhattar R, Berger SL (2001). Snf1--a histone kinase that works in concert with the histone acetyltransferase Gcn5 to regulate transcription. Science.

[99] Lee DY, Hayes JJ, Pruss D, Wolffe AP (1993). A positive role for histone acetylation in transcription factor access to nucleosomal DNA. Cell.

[100] Burkewitz K, Zhang Y, Mair WB (2014). AMPK at the nexus of energetics and aging. Cell Metab.

[101] Shimazu T, Hirschey MD, Newman J, He W, Shirakawa K, Le Moan N, Grueter CA, Lim H, Saunders LR, Stevens RD (2013). Suppression of oxidative stress by beta-hydroxybutyrate, an endogenous histone deacetylase inhibitor. Science.

[102] Yang W, Hong YH, Shen XQ, Frankowski C, Camp HS, Leff T (2001). Regulation of transcription by AMP-activated protein kinase: phosphorylation of p300 blocks its interaction with nuclear receptors. J Biol Chem.

[103] de Ruijter AJ, van Gennip AH, Caron HN, Kemp S, van Kuilenburg AB (2003). Histone deacetylases (HDACs): characterization of the classical HDAC family. Biochem J.

[104] Boffa LC, Vidali G, Mann RS, Allfrey VG (1978). Suppression of histone deacetylation in vivo and in vitro by sodium butyrate. J Biol Chem.

[105] Gottlicher M, Minucci S, Zhu P, Kramer OH, Schimpf A, Giavara S, Sleeman JP, Lo Coco F, Nervi C, Pelicci PG (2001). Valproic acid defines a novel class of HDAC inhibitors inducing differentiation of transformed cells. EMBO J.

[106] González A, Hall MN, Lin S-C, Hardie DG (2020). AMPK and TOR: The yin and yang of cellular nutrient sensing and growth control. Cell Metabolism.

[107] Ruderman NB, Xu XJ, Nelson L, Cacicedo JM, Saha AK, Lan F, Ido Y (2010). AMPK and SIRT1: a long-standing partnership?. Am J Physiol Endocrinol Metab.

[108] Banerjee J, Bruckbauer A, Zemel MB (2016). Activation of the AMPK/Sirt1 pathway by a leucine-metformin combination increases insulin sensitivity in skeletal muscle, and stimulates glucose and lipid metabolism and increases life span in Caenorhabditis elegans. Metabolism.

[109] Burns AR, Kwok TC, Howard A, Houston E, Johanson K, Chan A, Cutler SR, McCourt P, Roy PJ (2006). High-throughput screening of small molecules for bioactivity and target identification in Caenorhabditis elegans. Nat Protoc.

[110] Onken B, Driscoll M (2010). Metformin induces a dietary restriction-like state and the oxidative stress response to extend C. elegans Healthspan via AMPK, LKB1, and SKN-1. Plos One.

[111] Cabreiro F, Au C, Leung KY, Vergara-Irigaray N, Cocheme HM, Noori T, Weinkove D, Schuster E, Greene ND, Gems D (2013). Metformin retards aging in *C. elegans* by altering microbial folate and methionine metabolism. Cell.

[112] Burns AR, Wallace IM, Wildenhain J, Tyers M, Giaever G, Bader GD, Nislow C, Cutler SR, Roy PJ (2010). A predictive model for drug bioaccumulation and bioactivity in *Caenorhabditis elegans*. Nat Chem Biol.

[113] Longnus SL, Wambolt RB, Parsons HL, Brownsey RW, Allard MF (2003). 5-Aminoimidazole-4-carboxamide 1-beta-D-ribofuranoside (AICAR) stimulates myocardial glycogenolysis by allosteric mechanisms. Am J Physiol Regul Integr Comp Physiol.

[114] Bain J, Plater L, Elliott M, Shpiro N, Hastie CJ, McLauchlan H, Klevernic I, Arthur JS, Alessi DR, Cohen P (2007). The selectivity of protein kinase inhibitors: a further update. Biochem J.

[115] Pacholec M, Bleasdale JE, Chrunyk B, Cunningham D, Flynn D, Garofalo RS, Griffith D, Griffor M, Loulakis P, Pabst B, Qiu X, Stockman B, Thanabal V, Varghese A, Ward J, Withka J, Ahn K (2010). SRT1720, SRT2183, SRT1460, and resveratrol are not direct activators of SIRT1. J Biol Chem.

[116] Adams S, Pathak P, Shao H, Lok JB, Pires-daSilva A (2019). Liposome-based transfection enhances RNAi and CRISPR-mediated mutagenesis in non-model nematode systems. Sci Rep.

[117] Horsthemke B (2018). A critical view on transgenerational epigenetic inheritance in humans. Nat Commun.

[118] Stiernagle T: Maintenance of *C. elegans*. WormBook 2006:1-11. 10.1895/wormbook.1.101.1 PMC4781397 18050451

[119] Robles P, Turner A, Zuco G, Adams S, Paganopoulou P, Winton M, Hill B, Kache V, Bateson C, Pires da Silva A. Parental energy-sensing pathways control intergenerational offspring sex determination in the nematode Auanema freiburgensis. figshare. 2021. 10.6084/m9.figshare.14381744.v1. 10.1186/s12915-021-01032-1 PMC8130380 34001117

[120] Bishop NA, Guarente L (2007). Two neurons mediate diet-restriction-induced longevity in *C. elegans*. Nature.

[121] Zuco G, Kache V, Robles P, Chaudhuri J, Hill B, Bateson C, Pires da Silva A. Sensory neurons control heritable adaptation to stress through germline reprogramming. bioRxiv. 2018:406033. 10.1101/406033.

[122] Altschul SF, Madden TL, Schaffer AA, Zhang J, Zhang Z, Miller W, Lipman DJ (1997). Gapped BLAST and PSI-BLAST: a new generation of protein database search programs. Nucleic Acids Res.

[123] Swedlow JR: Chapter 17 - quantitative fluorescence microscopy and image deconvolution. In: Methods in Cell Biology. Edited by Sluder G, Wolf DE, vol. 114: Academic Press; 2013: 407-426. 10.1016/B978-0-12-407761-4.00017-8 23931516

[124] Swedlow JR, Hu K, Andrews PD, Roos DS, Murray JM. Measuring tubulin content in Toxoplasma gondii: a comparison of laser-scanning confocal and wide-field fluorescence microscopy. Proc Natl Acad Sci U S A. 2002;99(4):2014–9. 10.1073/pnas.022554999 PMC122311 11830634

[125] Phillips CM, McDonald KL, Dernburg AF (2009). Cytological analysis of meiosis in Caenorhabditis elegans. Methods Mol Biol.

[126] Jeong D-E, Lee Y, Lee S-JV, Huang LE (2018). Western blot analysis of C. elegans proteins. Hypoxia: Methods and Protocols.

[127] Ho J, Tumkaya T, Aryal S, Choi H, Claridge-Chang A (2019). Moving beyond P values: data analysis with estimation graphics. Nat Methods.

