# Supplementary material for: Parental energy-sensing pathways control intergenerational offspring sex determination in the nematode Auanema freiburgensis
Source: BMC Biol. 2021 May 17;19:102. doi: 10.1186/s12915-021-01032-1 (PMC8130380; doi:10.1186/s12915-021-01032-1)
Supplement: Supplementary file 1 — Additional file 1: Figure S1. STRAD and LKB1 antibody staining are higher in animals in crowding conditions. Figure S2. Metformin and trichostatin affect the F1 sex ratios in the absence of diluted CM. Figure S3. PTEN/DAF-18 in the germline is cytoplasmic and higher in non-crowding conditions. Figure S4. AKT pThr308 in the germline is nuclear and higher in crowding conditions. Figure S5. Acetylation in the germline is nuclear and higher in crowding conditions. Figure S6. Alignment of the protein sequences of the immunogens used to raise the antibodies and their predicted A. freiburgensis orthologs. Figure S7. Uncropped images of Western blots. [file 12915_2021_1032_MOESM1_ESM.pdf]

Parental energy-sensing pathways control intergenerational offspring sex determination in the nematode *Auanema freiburgensis*

Pedro Robles[1], Anisa Turner[1], Giusy Zuco[1], Sally Adams[1], Panagiota Paganopolou[1], Michael Winton[1], Beth Hill[1], Vikas Kache[2], Christine Bateson[2], Andre Pires-daSilva[*]

[1]School of Life Sciences University of Warwick, Coventry, CV4 7AL, UK

[2]Department of Biology, University of Texas at Arlington, Arlington, TX, 76019, USA

[*]Corresponding author

Supplementary material

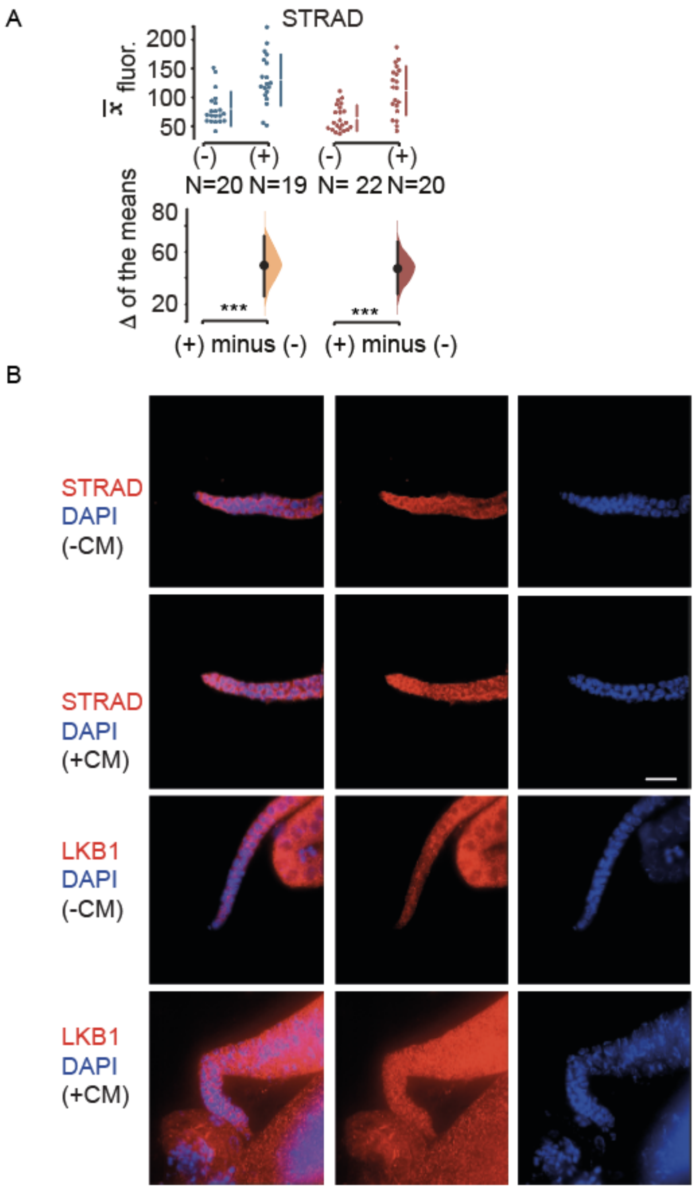

Additional file 1: Figure S1. STRAD and LKB1 antibody staining are higher in animals in crowding conditions. A. Mean antibody fluorescence ($\bar{x}$) in the pre-meiotic (blue) and meiotic portion (red) of the germline, in the absence (-) or presence (+) of conditioned medium. N= sample sizes. Graphical representation as Fig. 2, with ***= $p \leq 0.001$. **B.** LKB1 and STRAD in the germline. Staining for antibodies (in red) against LKB1 and STRAD of gonads dissected from hermaphrodites incubated in the presence of either (-) CM or (+) CM. The DNA was stained with DAPI (blue). Bar, 15 µm.

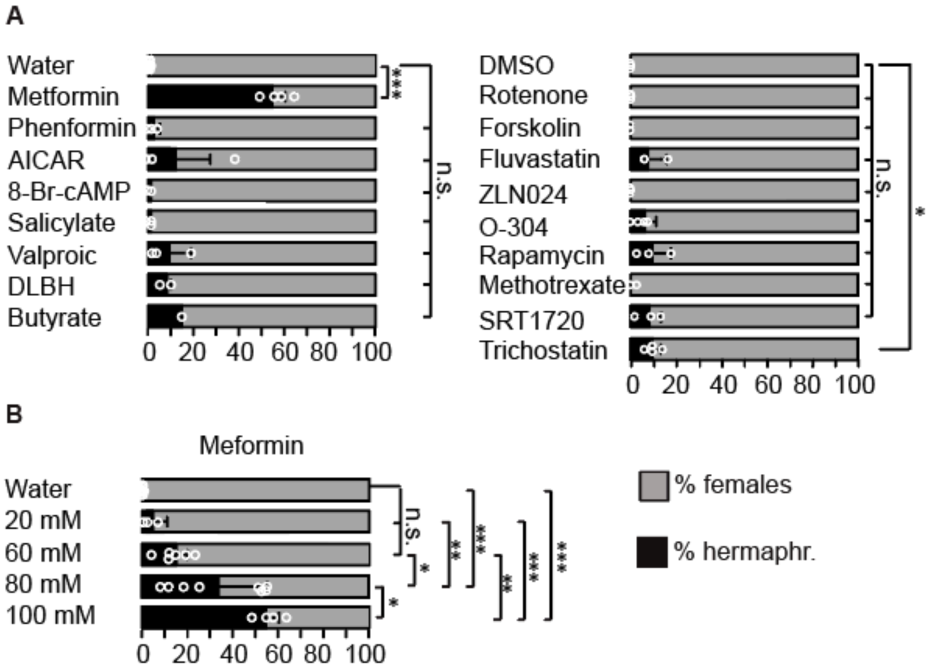

Additional file 1: Figure S2. Metformin and trichostatin affect the F1 sex ratios in the absence of diluted CM. A, B. Mean percentage and SD of hermaphrodite and female F1 offspring from hermaphrodites treated with chemicals. Chemicals were dissolved either in water (left) or in DMSO (right). **B.** Dose-dependent increase of F1 hermaphrodite production when using metformin. We performed one-way analysis of variance (ANOVA) followed by a post-hoc Holm-Sidak test for multiple comparisons. n.s., p>0.05; * = p ≤ 0.05; ** = p ≤ 0.01; ***= p ≤ 0.001

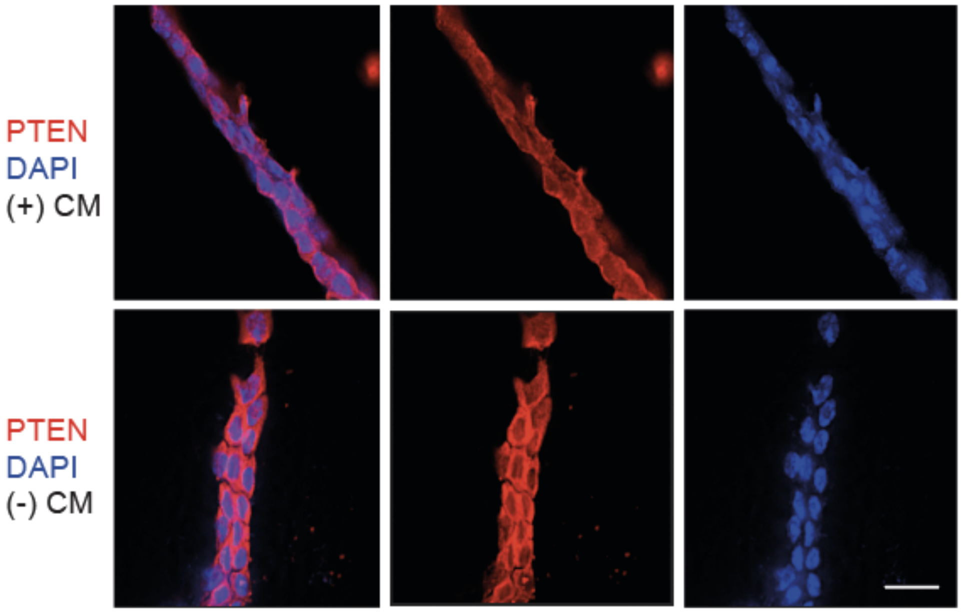

Additional file 1: Figure S3. PTEN/DAF-18 in the germline is cytoplasmic and higher in non-crowding conditions. Staining for antibodies (in red) against PTEN/DAF-18 of gonads dissected from hermaphrodites incubated in the presence of either (-) CM or (+) CM. The DNA was stained with DAPI (blue). Bar, 15 μm.

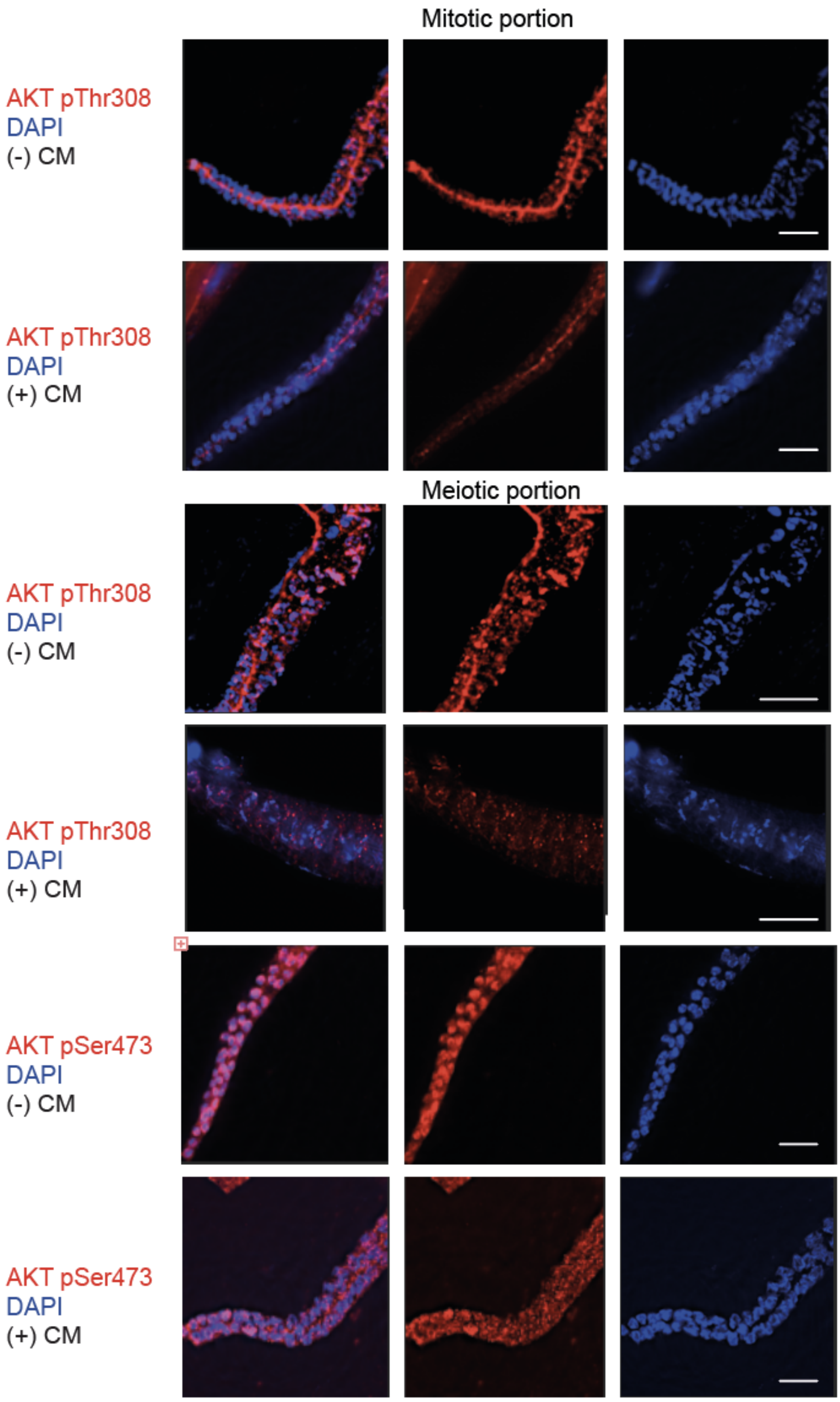

Additional file 1: Figure S4. AKT pThr308 in the germline is nuclear and higher in crowding conditions. Staining for antibodies (in red) against AKT pThr308 of gonads dissected from hermaphrodites incubated in the presence of either (-) CM or (+) CM. The DNA was stained with DAPI (blue). Bar, 15 µm.

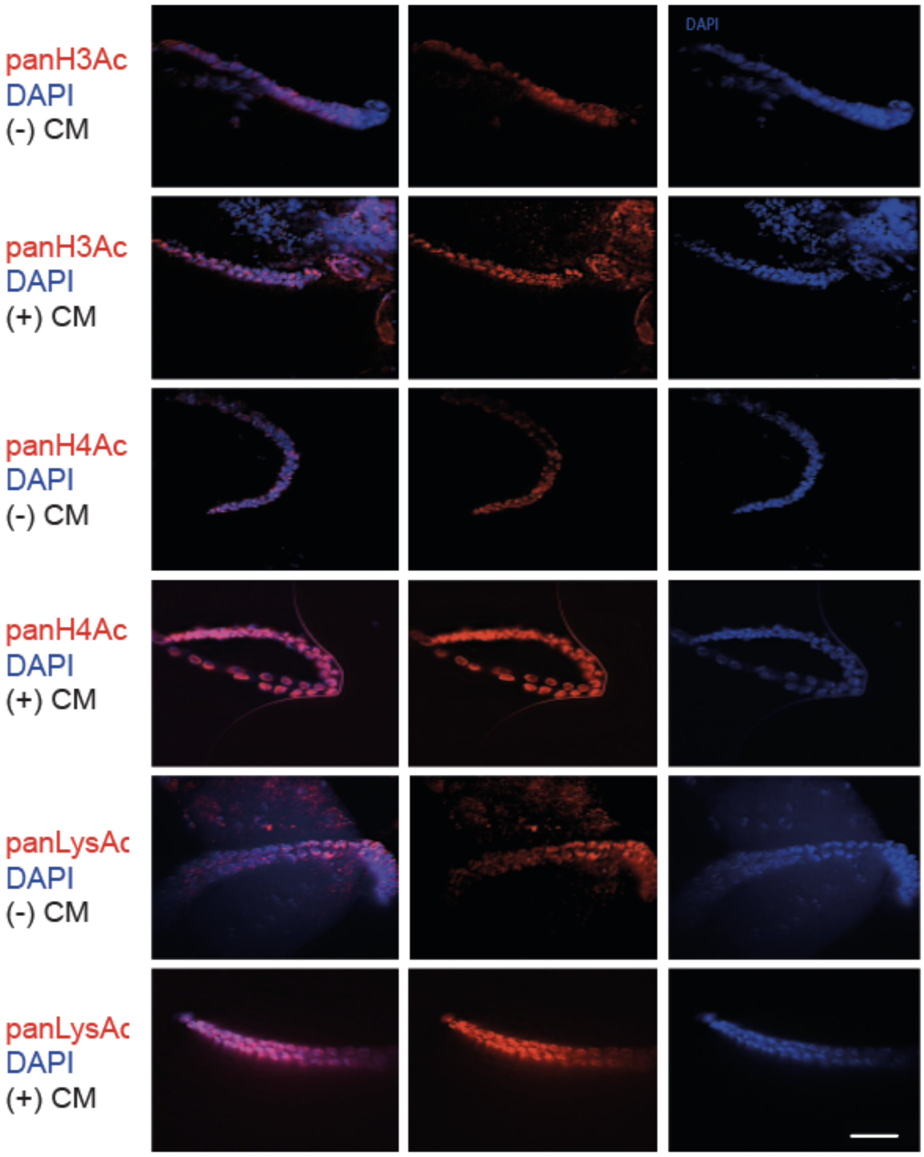

Additional file 1: Figure S5. Acetylation in the germline is nuclear and higher in crowding conditions. Staining for antibodies (in red) against panH3Ac, panH4Ac and panLysAc of gonads dissected from hermaphrodites incubated in the presence of either (-) CM or (+) CM. The DNA was stained with DAPI (blue). Bar, 15 μm.

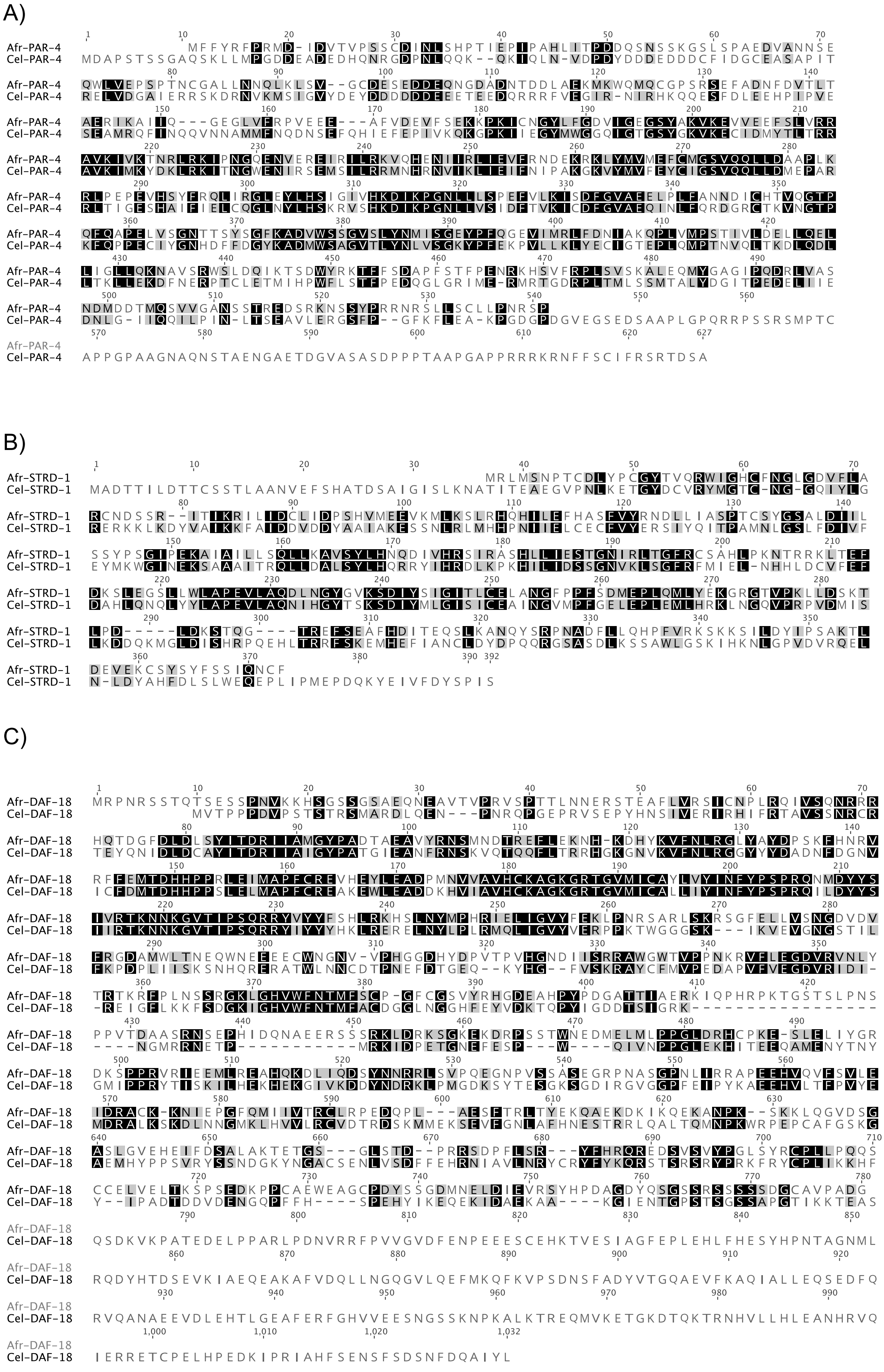
A)
Afr-PAR-4
Cel-PAR-4
B)
Afr-STRD-1
Cel-STRD-1
C)
Afr-DAF-18
Cel-DAF-18

D)

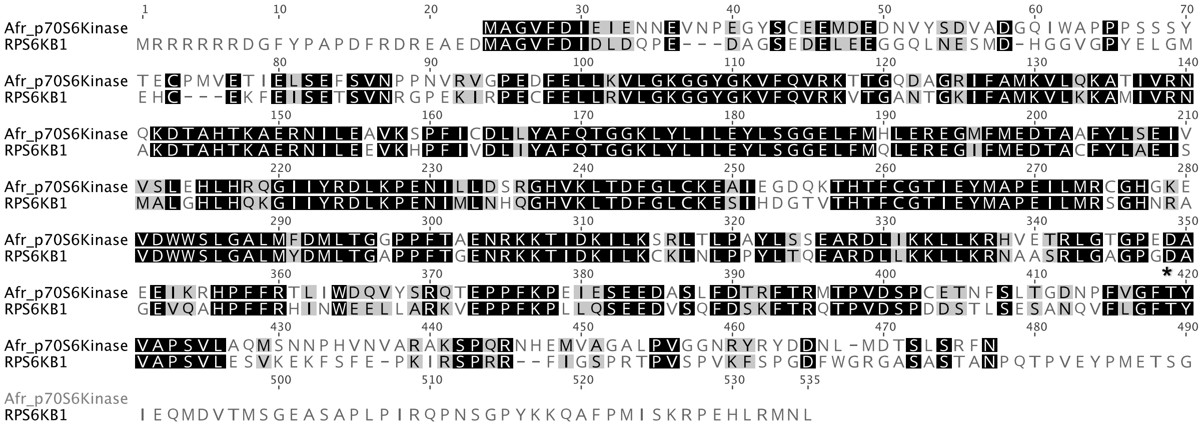
Afr_p70S6Kinase MAGVFDIEIENNEVNPEGYSCEEMDEDNVYSDVADGQIWAPPPSSSY
RPS6KB1 MRRRRRRDGFYPAPDFRDREAEDMAGVFDIDLDQPE---DAGSEDELEEGGQLNESMD-HGGVGPYELGM
Afr_p70S6Kinase TECPMVETIELSEFSVNPPNVRVGPEDFELLKVLGKGGYGKVFQVRKTTGQDAGRIFAMKVLQKATIVRN
RPS6KB1 EHC---EKFEISETSVNRGPEKIRPECFELLRVLGKGGYGKVFQVRKVTGANTGKIFAMKVLKKAMIVRN
Afr_p70S6Kinase QKDTAHTKAERNILEAVKSPFICDLLYAFQTGGKLYLILEYLSGGELFMHLEREGMFMEDTAAFYLSEIV
RPS6KB1 AKDTAHTKAERNILEEVKHPFIVDLIYAFQTGGKLYLILEYLSGGELFMQLEREGIFMEDTACFYLAEIS
Afr_p70S6Kinase VSLEHLHRQGIIYRDLKPENILLDSRGHVKLTDFGLCKEAIEGDQKTHTFCGTIEYMAPEILMRCGHGKE
RPS6KB1 MALGHLHQKGIIYRDLKPENIMLNHQGHVKLTDFGLCKESIHDGTVTHTFCGTIEYMAPEILMRSGHNRA
Afr_p70S6Kinase VDWWSLGALMFDMLTGGPPFTAENRKKTIDKILKSRLTLPAYLSSEARDLIKKLLKRHVETRLGTGPEDA
RPS6KB1 VDWWSLGALMYDMLTGAPPFTGENRKKTIDKILKCKLNLPPYLTQEARDLLKKLLKRNAASRLGAGPGDA
Afr_p70S6Kinase EEIKRHPFFRTLIWDQVYSRQTEPPFKPEIESEEDASLFDTRFTRMTPVDSPCETNFSLTGDNPFVGFTY
RPS6KB1 GEVQAHPFFRHINWEELLARKVEPPFKPLLQSEEDVSQFDSKFTRQTPVDSPDDSTLSESANQVFLGFTY
Afr_p70S6Kinase VAPSVLAQMSNNPHVNVARAKSPQRNHEMVAGALPVGGNRYRYDDNL-MDTSLSRFN
RPS6KB1 VAPSVLESVKEKFSFE-PKIRSPRR--FIGSPRTPVSPVKFSPGDFWGRGASASTANPQTPVEYPMETSG
Afr_p70S6Kinase
RPS6KB1 IEQMDVTMSGEASAPLPIRQPNSGPYKKQAFPMISKRPEHLRMNL

E)

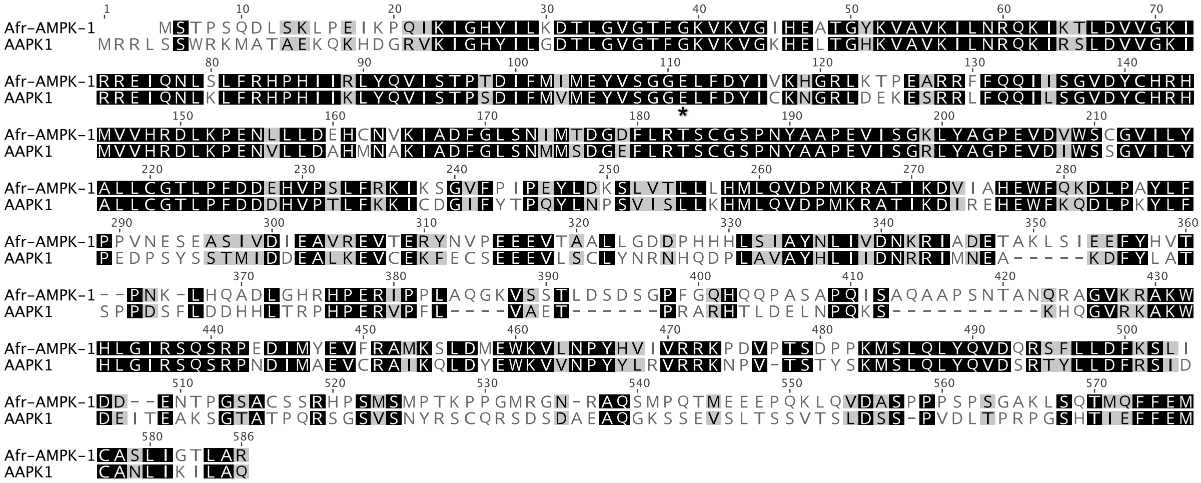
Afr-AMPK-1 MSTPSQDLSKLPEIKPQIKIGHYILKDTLGVGTFGKVKVGIHEATGYKVAVKILNRQKIKTLDVVGKI
AAPK1 MRRLSSWRKMATAEKQKHDGRVKIGHYILGDTLGVGTFGKVKVGKHELTGHKVAVKILNRQKIRSLDVVGKI
Afr-AMPK-1 RREIQNLSLFRHPHIIRLYQVISTPTDIFMIMEYVSGGELFDYIVKHGRLKTPEARRFFQQIISGVDYCHRH
AAPK1 RREIQNLKLFRHPHIIKLYQVISTPSDIFMVMEYVSGGELFDYICKNGRLDEKESRRLFQQILSGVDYCHRH
Afr-AMPK-1 MVVHRDLKPENLLLDEHCNVKIADFGLSNIMTDGDFLRTSCGSPNYAAPEVISGKLYAGPEVDVWSCGVILY
AAPK1 MVVHRDLKPENVLLDAHMNAKIADFGLSNMMSDGEFLRTSCGSPNYAAPEVISGRLYAGPEVDIWSSGVILY
Afr-AMPK-1 ALLCGTLPFDDEHVPSLFRKIKSGVFPIPEYLDKSLVTLLLHMLQVDPMKRATIKDVIAHEWFQKDLPAYLF
AAPK1 ALLCGTLPFDDDHVPTLFKKICDGIFYTPQYLNPSVISLLKHMLQVDPMKRATIKDIREHEWFKQDLPKYLF
Afr-AMPK-1 PPVNESEASIVDIEAVREVTERYNVPEEEVTAALLGDDPHHHLSIAYNLIVDNKRIADETAKLSIEEFYHVT
AAPK1 PEDPSYSSTMIDDEALKEVCEKFECSEEEVLSCLYNRNHQDPLAVAYHLIIDNRRIMNEA-----KDFYLAT
Afr-AMPK-1 --PNK-LHQADLGHRHPERIPPLAQGKVSSTLDSDSGPFGQHQQPASAPQISAQAAPSNTANQRAGVKRAKW
AAPK1 SPPDSFLDDHHLTRPHPERVPFL----VAET------PRARHTLDELNPQKS----------KHQGVRKAKW
Afr-AMPK-1 HLGIRSQSRPEDIMYEVFRAMKSLDMEWKVLNPYHVIVRRKPDVPTSDPPKMSLQLYQVDQRSFLLDFKSLI
AAPK1 HLGIRSQSRPNDIMAEVCRAIKQLDYEWKVVNPYYLRVRRKNPV-TSTYSKMSLQLYQVDSRTYLLDFRSID
Afr-AMPK-1 DD--ENTPGSACSSRHPSMSMPTKPPGMRGN-RAQSMPQTMEEPQKLQVDASPPPSPSGAKLSQTMQFFEM
AAPK1 DEITEAKSGTATPQRSGSVSNYRSCQRSDSDAEAQGKSSEVSLTSSVTSLDSS-PVDLTPRPGSHTIEFFEM
Afr-AMPK-1 CASLIGTLAR
AAPK1 CANLIKILAQ

F)

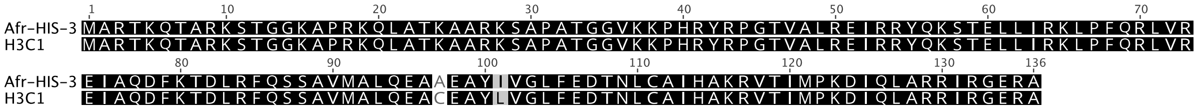
Afr-HIS-3 MARTKQTARKSTGGKAPRKQLATKAARKSAPATGGVKKPHRYRPGTVALREIRRYQKSTELLIRKLPFQRLVR
H3C1 MARTKQTARKSTGGKAPRKQLATKAARKSAPATGGVKKPHRYRPGTVALREIRRYQKSTELLIRKLPFQRLVR
Afr-HIS-3 EIAQDFKTDLRFQSSAVMALQEAAEAYIVGLFEDTNLCAIHAKRVTIMPKDIQLARRIRGERA
H3C1 EIAQDFKTDLRFQSSAVMALQEACEAYLVGLFEDTNLCAIHAKRVTIMPKDIQLARRIRGERA

G)

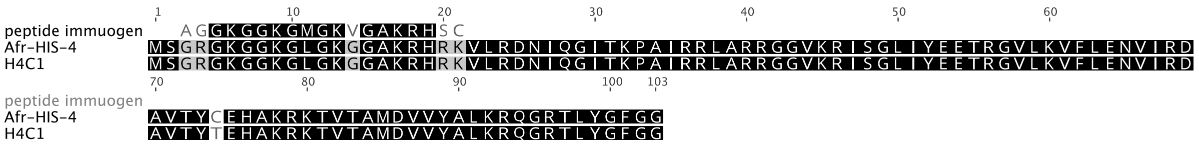
peptide immuogen AGGKGGKGMGKVGAKRHSC
Afr-HIS-4 MSGRGKGGKGLGKGGAKRHRKVLRDNIQGITKPAIRRLARRGGVKRISGLIYEETRGVLKVFLENVIRD
H4C1 MSGRGKGGKGLGKGGAKRHRKVLRDNIQGITKPAIRRLARRGGVKRISGLIYEETRGVLKVFLENVIRD
peptide immuogen
Afr-HIS-4 AVTYCEHAKRKTVTAMDVVYALKRQGRTLYGFGG
H4C1 AVTYTEHAKRKTVTAMDVVYALKRQGRTLYGFGG

H)

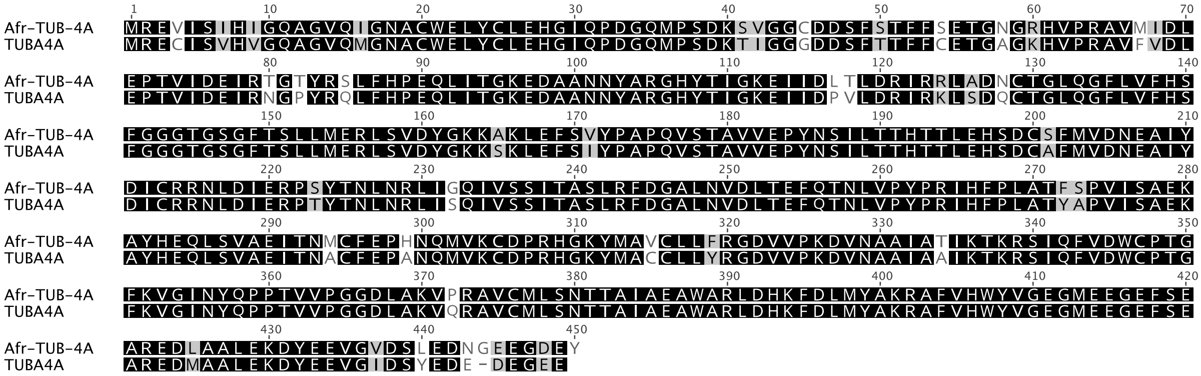

I)

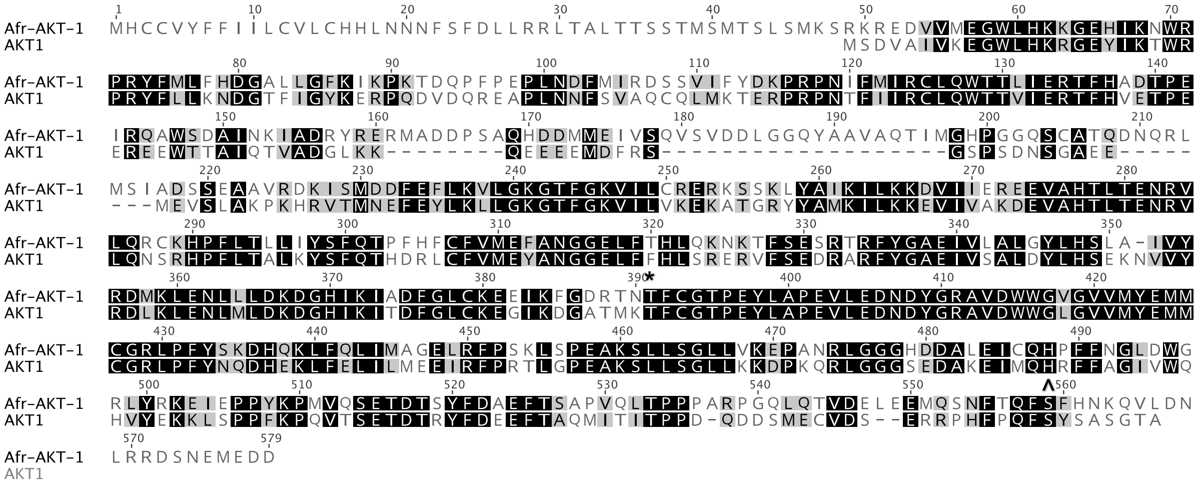

Additional file 1: Figure S6. Alignment of the protein sequences of the immunogens used to raise the antibodies and their predicted *A. freiburgensis* orthologs. Transcript translation and alignments of the protein sequences were performed with the Geneious software (v_10.0.9). Alignment of the *C. elegans* proteins (**A**) *Cel*-PAR-4 (**B**) *Cel*-STRD-1 and (**C**) *Cel*-DAF-18 and the *H. sapiens* proteins (**D**) RPS6KB1 (**E**) AAPK1 (**F**) H3C1 (**G**) H4C1 (**H**) TUB4A and (**I**) AKT1 with their predicted *A. freiburgensis* orthologs. Key amino acids are conserved in Afr-p70S6Kinase (*Thr389), Afr-AMPK-1 (*Thr172) and Afr-AKT-1 (*Thr308 and ^ Ser473). The pan-Acetyl H4 antibody (Active Motif) was raised against a short, acetylated peptide immunogen (**I**) but the antibody is used routinely to detect acetylated H4 in human cell lines. The *Afr*-HIS-4 ortholog shows full homology to the targeted region in the *H. sapiens* HC41 and conservation of all the K residues with the peptide immunogen.

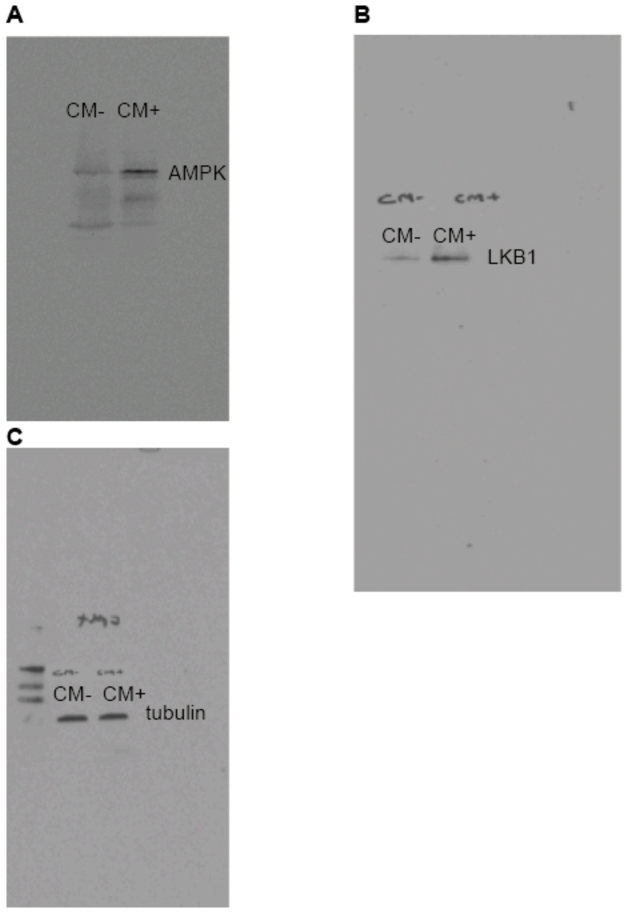

Additional File 1: Figure S7. Uncropped images of Western blots. (A) AMPK pThr172 antibody, **(B)** LKB1 antibody and **(C)** tubulin antibody. They correspond to Figure 2D.
